# Progress in the Synthesis of Organoselenium Compounds: Conventional Routes Versus Green Approaches

**DOI:** 10.3390/molecules31152674

**Published:** 2026-07-31

**Authors:** Chintankumar Padariya, Anita Kornicka

**Affiliations:** Department of Chemical Technology of Drugs, Faculty of Pharmacy, Medical University of Gdańsk, Al. Gen. J. Hallera 107, 80-416 Gdańsk, Poland; anita.kornicka@gumed.edu.pl

**Keywords:** catalytic methodologies, green synthesis, organoselenium compounds, sustainable chemistry, therapeutic applications

## Abstract

Organoselenium chemistry has progressed from the early synthesis of simple selenoorganic molecules in the 20th century to advanced methodologies aligned with the principles of green chemistry. Conventional synthetic approaches, frequently dependent on hazardous reagents and organic solvents, are increasingly being replaced by environmentally benign strategies, including solvent-free reactions, aqueous and bio-based solvent systems, microwave-assisted synthesis, and mechanochemical techniques. These sustainable methodologies offer significant advantages, such as enhanced reaction efficiency, higher or comparable yields, reduced waste generation, improved safety, and lower environmental impact. In parallel, evolving regulatory standards and industrial practices are encouraging the adoption of greener synthetic protocols to minimize hazardous waste and support safer pharmaceutical manufacturing. This review systematically categorizes organoselenium compounds, highlighting their synthetic methodologies, structural characteristics, and biological activities. Overall, recent advances emphasize the therapeutic potential of organoselenium compounds and demonstrate the essential role of sustainable synthetic chemistry in the development of future medicinal agents.

## 1. Introduction

Organic chemistry has elevated the development of sustainable and environmentally benign synthetic methodologies in response to the rising need to reduce the ecological footprint of chemical processes [1,2,3]. In this context, organoselenium compounds have attracted sustained interest owing to their distinctive reactivity, redox behavior, and broad biological relevance, particularly in medicinal and synthetic chemistry [4,5,6,7,8,9]. Despite their importance, conventional routes to organoselenium compounds often rely on multistep protocols and toxic reagents, raising significant environmental and practical concerns [10,11,12]. Recent advances in asymmetric synthesis, catalysis, and the adoption of green solvents have opened new opportunities for developing efficient, sustainable, and eco-friendly synthetic approaches to organoselenium compounds [13,14,15].

**Figure 1:**
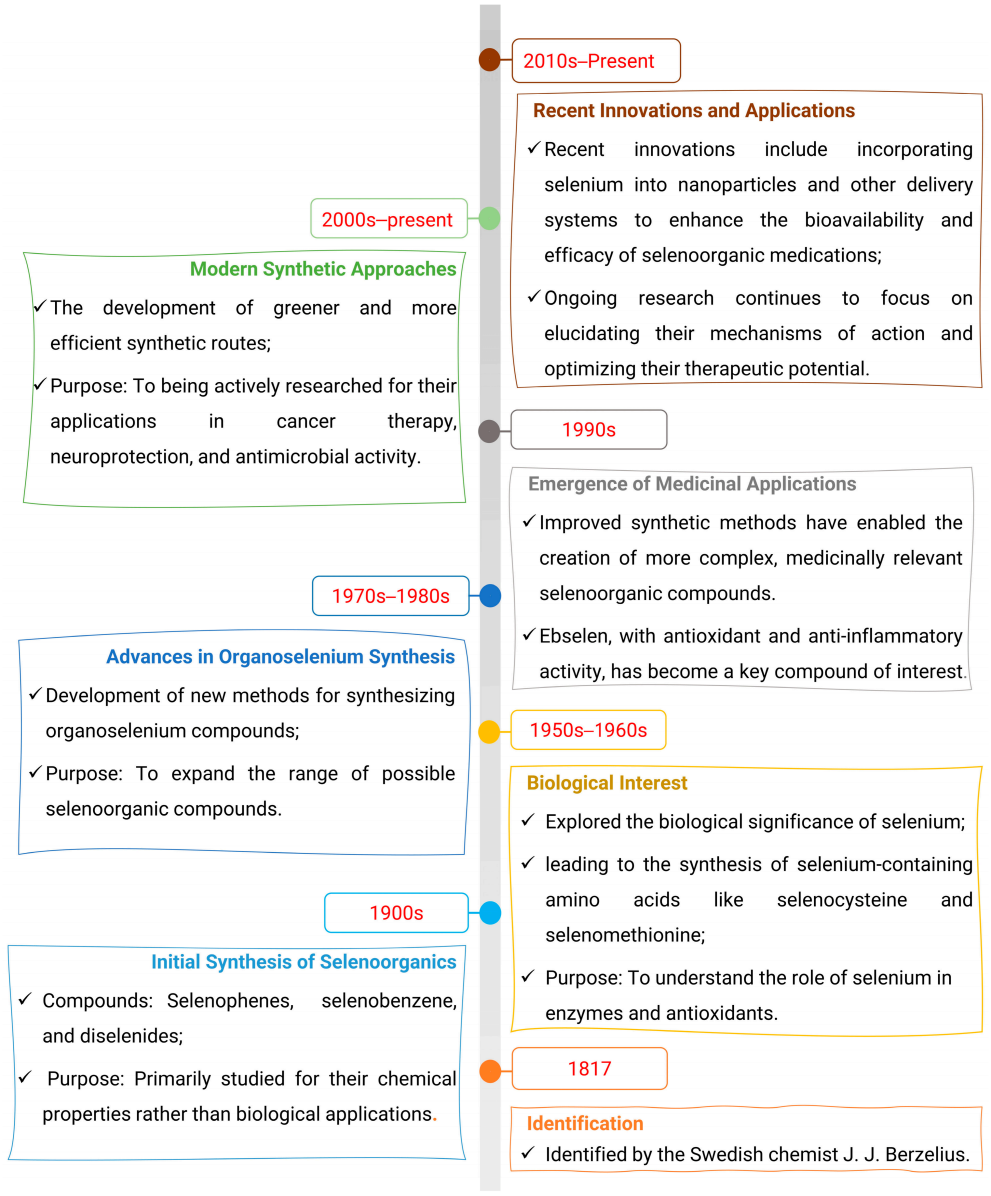
The historical graph depicting the progress in the synthesis of organoselenium compounds.

The progress in the syntheses of organoselenium compounds is summarized in Figure 1. The early 20th century marked the initial syntheses of simple organoselenium molecules, including selenophenes, selenobenzene, and diselenides. These compounds exhibited unique aromaticity, enhanced nucleophilicity, and distinctive redox properties relative to their sulfur and oxygen analogues, providing early insights into the bonding behavior of selenium within organic frameworks [16]. At this stage, research primarily focused on fundamental chemical properties, with limited attention to biological applications [17].

The biological significance of selenium became increasingly apparent in the mid-20th century through the synthesis of selenium-containing amino acids such as selenocysteine (SeCys) and selenomethionine (SeMet). These discoveries supported a role for selenium in enzymatic function and antioxidant defense mechanisms [18]. Since the 1970s, organoselenium synthesis has expanded rapidly with the introduction of new reagents and synthesis strategies, including selenenyl halides, nucleophilic substitutions, electrophilic additions, and oxidative cyclizations. These methodologies enabled the preparation of diverse selenium-functionalized structures, such as seleniranes, selenoepoxides, and heterocycles, including selenophenes and selenazoles. Additionally, selenium-specific reagents and radical-based strategies further broadened the scope of carbon–selenium bond formation [19].

By the 1990s, synthetic trends had advanced to structurally complex organoselenium compounds with potential therapeutic applications. Notably, ebselen progressed to clinical trials for the treatment of stroke, highlighting the translational relevance of organoselenium chemistry despite regulatory challenges [20]. Since 2000, the field has increasingly embraced sustainability-oriented methodologies, including transition-metal catalysis, biomimetic strategies, and the use of environmentally benign reagents. These approaches have facilitated more efficient and selective synthesis while enabling applications in oncology, neuroprotection, and antimicrobial therapy [7,15,21,22].

Conventional synthetic routes, while effective, often relied on hazardous reagents, toxic solvents, and energy-intensive processes, raising environmental and safety concerns. The adoption of green chemistry has enabled the development of more sustainable, efficient, and safer synthetic strategies, including solvent-free reactions, aqueous and bio-based solvents, mechanochemical and microwave-assisted techniques, and electrochemical methods. Concurrently, green chemistry has emerged as a central framework for sustainable innovation in pharmaceutical synthesis. Green chemistry integrates lifecycle considerations from molecular design to end-of-life disposal to minimize environmental and human health risks. Given that the pharmaceutical industry is a major contributor to chemical waste, largely due to solvent usage, green chemistry principles have been progressively adopted to enhance process efficiency and reduce hazardous emissions. Notably, solvent waste constitutes a substantial fraction of active pharmaceutical ingredient (API) production, driving regulatory and industrial efforts toward safer alternatives and waste minimization strategies [23].

Organoselenium synthesis has focused on methods that are highly selective and catalytic, as well as the efficient utilization of resources, in order to avoid the use of hazardous chemicals, reduce solvent usage, and minimize waste production during purification [23,24]. The integration of green chemistry into organoselenium synthesis has enabled greener synthetic strategies, including solvent-free transformations, aqueous media and bio-derived solvents, mechanochemical and microwave-assisted processes, and electrochemical methodologies. These approaches improve synthesis efficiency by reducing energy consumption, minimizing waste generation, and promoting the use of safer selenium sources.

The choice of solvents remains an essential factor, with greener choices such as water, ethanol, biodegradable solvents, ionic liquids (ILs), and supercritical carbon dioxide (scCO_2_) being studied despite limitations in cost, recycling, and scalability. Solvent assessment is aided by instruments such as the United States Environmental Protection Agency’s Green Chemistry Expert System [25]. Furthermore, solvent-minimized approaches, including mechanochemical and electrochemical methods, offer sustainable routes for selenium bond formation under milder conditions.

The current review conducted a thorough search of the Scopus, ScienceDirect, and Web of Science databases for material published since 1982. The following keywords were used to search for articles relevant to the topic: “organoselenium compounds,” “selenium-containing compounds,” “green synthesis,” “sustainable synthesis,” “selenium incorporation,” “solvent-free synthesis,” “mechanochemistry,” “electrochemical synthesis,” and “microwave assisted synthesis.” Research articles on sustainable selenium sources, green conditions, novel solvents, and energy-saving methods were reviewed, whereas irrelevant papers were omitted from the review.

Based on the selected literature, representative conventional and green synthetic routes for organoselenium compounds are discussed individually and comparatively assessed using a unified framework that considers reaction-specific parameters, including solvent selection, reaction conditions, reaction time, reagent/catalyst utilization, yield, and overall sustainability. This approach provides a systematic understanding of the transition from traditional synthetic practices toward greener strategies and highlights future opportunities for developing environmentally responsible methodologies in organoselenium chemistry.

### Organoselenium Compounds: Therapeutic Roles

Selenium is an essential trace element that plays a pivotal role in maintaining cellular redox homeostasis, primarily through its incorporation into antioxidant enzymes such as glutathione peroxidases (GPx) and thioredoxin reductase (TrxR) [26]. These selenoenzymes protect biological systems against oxidative damage by catalyzing the reduction of hydrogen peroxide, lipid hydroperoxides, and other reactive oxygen species (ROS) [27,28,29].

**Figure 2:**
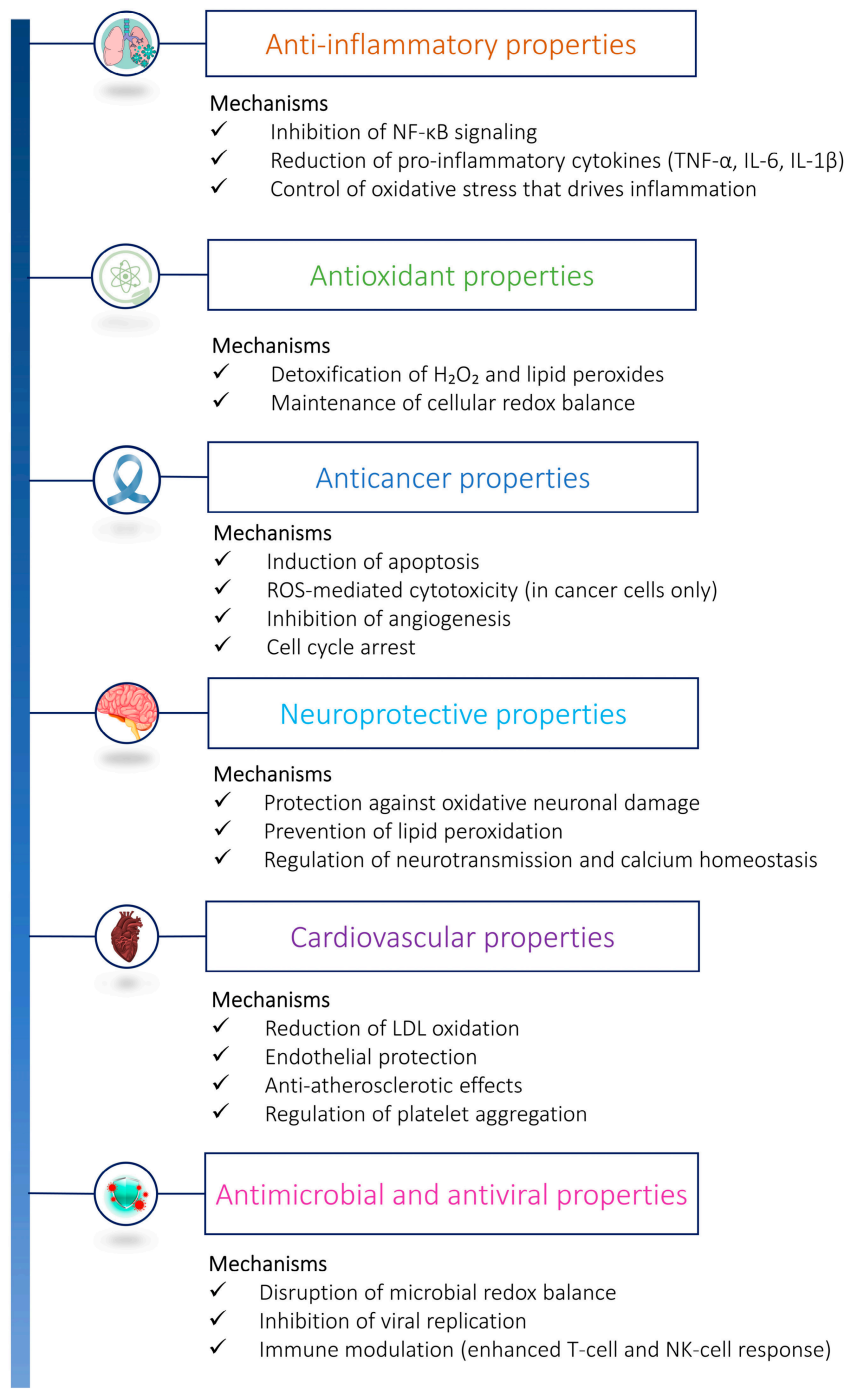
Organoselenium compounds and their significance in medicine.

Organoselenium chemistry has been extensively explored, with small-molecule compounds demonstrating GPx-like activity, radical scavenging, and modulation of redox-sensitive signaling pathways, thereby acting as effective redox modulators with therapeutic potential across multiple disease models and biological pathways [27,30,31,32,33,34]. Figure 2 illustrates the diverse health benefits of organoselenium compounds, highlighting their expanding significance in medical science, encompassing antioxidant, anti-inflammatory, anticancer, neuroprotective, cardiovascular, and antimicrobial effects. Moreover, detailed information on the structures, biological activities, study types, and molecular or pharmacological targets of organoselenium compounds is provided in the Supplementary Material (Table S1) [35,36,37,38,39,40,41,42,43,44,45,46,47,48,49,50,51,52,53,54,55,56,57,58].

The antioxidant activity of organoselenium compounds is fundamentally rooted in the redox chemistry of selenium, particularly in functional groups capable of cycling between reduced and oxidized states. Selenols, selenenic acids, selenoxides, and diselenides undergo reversible redox reactions, detoxifying hydrogen peroxide and lipid peroxides while maintaining cellular redox balance. These redox cycles are analogous to those seen in native GPx enzymes, albeit generally with lower catalytic efficiency [32,34]. SeCys is vital in therapeutic applications owing to its distinctive redox characteristics and its incorporation into important selenoproteins such as GPx and TrxR [59,60]. SeMet exhibits strong antioxidant properties, helps modulate immune responses, and plays a crucial role in thyroid hormone metabolism through the activity of selenium-dependent enzymes [60]. The anti-inflammatory effects of organoselenium compounds are closely linked to their antioxidant activity and modulation of redox-sensitive signaling pathways, particularly through inhibition of NF-κB activation and downstream cytokine expression.

Selenium species and organoselenium compounds, such as ebselen, can interact with redox-sensitive cysteine residues in regulatory proteins. This interaction inhibits NF-κB signaling and reduces the production of pro-inflammatory cytokines, including TNF-α, IL-6, and IL-1β, in immune cells and inflammatory models. As a result, ROS-mediated inflammatory cascades are suppressed and redox homeostasis is restored. For example, ebselen decreases LPS-induced NF-κB nuclear translocation and TNF-α production in Kupffer cells [55,61]. Selenium and organoselenium supplementation have also been shown to mitigate inflammation via modulation of MAPK and NF-κB pathways and enhance antioxidant enzyme activities such as GPx, decreasing lipid peroxidation and inflammatory signaling in ischemia–reperfusion and colitis models [62,63]. Additionally, selenium influences inflammasome activation; selenium compounds reduce NLRP3 inflammasome activation and pro-inflammatory signaling cascades, supporting the resolution of inflammation in chronic inflammatory conditions [64,65]. This results in reduced production of pro-inflammatory cytokines, including TNF-α, IL-6, and IL-1β [66,67]. Selenols and selenocyanates, through their high nucleophilicity, can scavenge reactive oxygen and nitrogen species (ROS/RNS) that otherwise perpetuate inflammatory cascades [68]. By controlling oxidative stress at both the molecular and cellular levels, these selenium functional groups suppress inflammation and promote restoration of redox homeostasis [61,69].

Organoselenium compounds selectively disrupt redox homeostasis in cancer cells. Seleninic acids (e.g., methaneseleninic acid), selenoketones, and selenocyanates act as redox-active donors, elevating ROS beyond the cytotoxic threshold in tumor cells [60,70]. Furthermore, organoselenium compounds including ebselen and its analogues can inhibit TrxR and other redox enzymes commonly upregulated in cancer, disrupting antioxidant defenses and sensitizing cancer cells to oxidative stress–induced death pathways and chemotherapeutics, with synergistic effects demonstrated when selenium compounds are combined with conventional drugs and nanoparticles to enhance anticancer efficacy and reduce systemic toxicity [71,72]. Studies suggest that SeMet may have chemopreventive effects by protecting DNA from oxidative damage and supporting apoptosis in abnormal cells. It also contributes to cardiovascular and cognitive health, with ongoing research into its potential benefits in neurodegenerative diseases such as Alzheimer’s disease. Due to its high absorption rate and low toxicity compared to inorganic selenium forms, SeMet is widely used in nutritional supplements and functional foods [53,73,74,75,76]. SeCys exhibits antioxidant, anticancer, and anti-inflammatory activities, making it a promising candidate for treating neurological disorders, cancer, and viral infections. The ability of SeCys to neutralize ROS and modulate immunological responses underscores its potential in neuroprotection and cardiovascular health in therapeutic applications [31,60,77]. Nevertheless, obstacles including toxicity at high doses, chemical instability, and inadequate bioavailability restrict their extensive application, prompting investigation into nanoparticle delivery advances and bioengineered selenoproteins for safer and more effective therapies [78,79].

Organoselenium compounds mitigate oxidative damage in neural tissue through multiple mechanisms. Selenols, selenoxides, diselenides, and selenium heterocycles (e.g., ebselen) reduce lipid peroxidation, maintain calcium homeostasis, and protect redox-sensitive ion channels and neurotransmitter systems. Seleniranes, due to their strained three-membered rings, can undergo controlled ring-opening reactions, enabling targeted neuronal redox modulation [54,80]. These mechanisms contribute to protection against neurodegenerative diseases such as Alzheimer’s disease and Parkinson’s disease, highlighting the potential of selenium-based therapeutics in cognitive and neuroprotective interventions.

The cardiovascular benefits of organoselenium compounds stem from their ability to reduce oxidative stress, preserve endothelial function, and maintain vascular integrity, in part through their incorporation into redox-active selenoproteins that mitigate reactive oxygen species and inhibit lipid peroxidation and endothelial dysfunction in atherosclerosis models [71,81]. Selenols and selenoxides have been linked to decreased low-density lipoprotein (LDL) oxidation and improved nitric oxide bioavailability, which support endothelial homeostasis and vasorelaxation, and selenium status has been shown to correlate inversely with markers of vascular oxidative damage and plaque formation [31,82].

Organoselenium functional groups simultaneously disrupt microbial redox homeostasis and can impair thiol-dependent enzymes critical for pathogen survival, as evidenced by the broad antimicrobial activity of organoselenium scaffolds that act through redox dyshomeostasis and thiol reactivity in bacterial and fungal cells [83,84]. Selenocyanates, selenoureas, and selenones readily react with thiol-containing proteins in bacteria, fungi, and viruses, impairing replication and metabolic function [83]. Compounds such as ebselen and related derivatives demonstrate potent antibacterial and antiviral activity, in part by covalent modification of active-site cysteine residues and inhibition of essential redox enzymes in pathogens, and have shown efficacy against drug-resistant bacteria and fungi such as *Candida* species, as well as antiviral effects including in vitro inhibition of SARS-CoV-2 proteases [56,57,85,86]. Moreover, selenium-containing agents can influence host immune function; selenium nutrition has been associated with enhanced lymphocyte proliferative responses and natural killer (NK) cell activity, suggesting a role in promoting T-cell and NK-cell responses against infectious agents, though clinical data vary with dose and status [87,88].

Organoselenium compounds exhibit notable anticancer activity, which arises from their capacity to selectively perturb redox homeostasis in tumor cells [89,90]. Seleninic acids such as methaneseleninic acid, as well as selenoketones and selenocyanates, can act as redox-active selenium donors that increase intracellular reactive oxygen species beyond the tolerance threshold of cancer cells. This pro-oxidant shift induces apoptosis, cell cycle arrest, and inhibition of angiogenesis [91]. Selenoureas and selenoamides have also been shown to interact with redox enzymes such as TrxR, leading to disruption of cancer cell survival pathways [92]. Importantly, the differential redox sensitivity of cancer versus normal cells allows selenium functional groups to exert cytotoxic effects selectively in malignant tissues [93].

The neuroprotective properties of organoselenium compounds are primarily associated with their ability to limit oxidative damage in neural tissue [32]. Selenols, selenoxides, and diselenides efficiently suppress lipid peroxidation and reduce accumulation of reactive oxygen species in neurons [94]. Selenium heterocycles such as ebselen modulate calcium homeostasis and neurotransmission by protecting redox-sensitive ion channels and receptors. Seleniranes, owing to their strained three-membered rings, can undergo controlled ring-opening reactions with thiols, enabling targeted redox modulation in neuronal environments [32]. Through these mechanisms, selenium functional groups help preserve neuronal integrity and counteract oxidative and inflammatory processes implicated in neurodegenerative disorders [58].

Cardiovascular benefits associated with organoselenium compounds arise from the collective actions of their functional groups on oxidative stress and vascular function [91]. Selenols and selenoxides inhibit LDL oxidation, a critical early event in atherogenesis [93]. Selenium-containing amides and heterocycles promote endothelial protection by maintaining nitric oxide bioavailability and reducing oxidative injury to vascular cells [91]. In addition, certain selenium species regulate platelet aggregation through thiol-dependent mechanisms, contributing to anti-atherosclerotic and antithrombotic effects [93]. These properties underscore the role of selenium redox chemistry in preserving cardiovascular homeostasis [91].

## 2. Organoselenium Compounds: Classification and Synthetic Approaches

**Figure 3:**
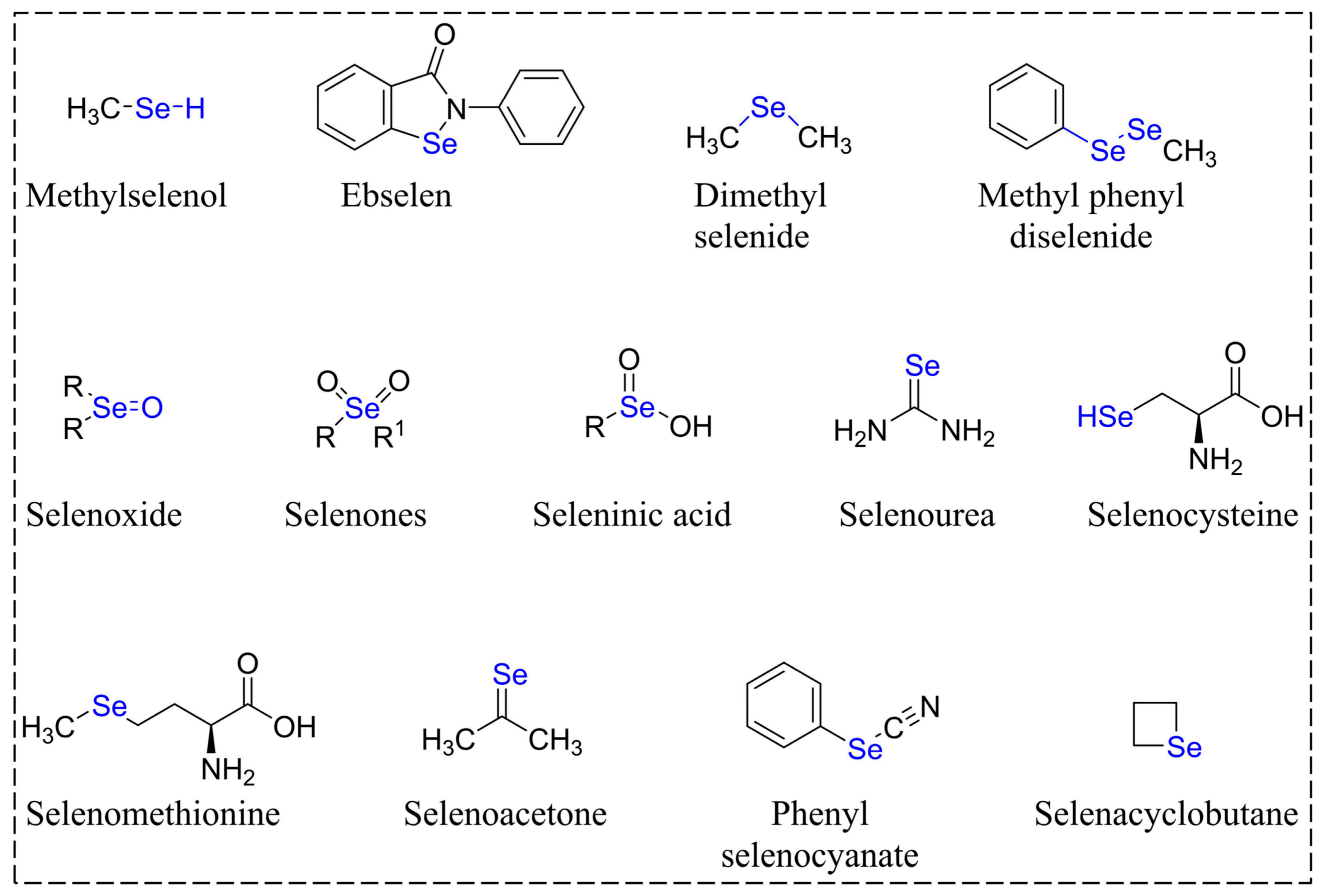
Structures of organoselenium compounds.

Organoselenium compounds encompass a diverse array of structural motifs and chemical properties that closely parallel those of their oxygen- and sulfur-containing counterparts, as illustrated in Figure 3, due to their shared chalcogen nature and analogous bonding patterns. The distinctive reactivity of these compounds originates from the unique physicochemical characteristics of selenium as a group 16 element, particularly its greater polarizability and lower electronegativity relative to sulfur and oxygen, which impart characteristic bonding patterns and reactivity profiles. In this review, organoselenium compounds are classified according to their structural analogs and classes of compounds, as summarized in Table 1, following established classifications of major organoselenium functional groups [95,96].

In addition to selenols, organoselenium compounds encompass a broad range of functional classes that parallel common oxygen- and sulfur-containing molecules, with selenides and diselenides serving as key dipolar analogs of ethers and disulfides, respectively. Selenides and diselenides resemble ethers and disulfides, respectively, and serve as important intermediates in organic synthesis, with diaryl diselenides widely used as precursors to electrophilic and nucleophilic selenium species in modern synthetic methodologies [15,107].

Oxidized derivatives such as selenoxides, selenones, and seleninic acids mimic sulfoxides, sulfones, and sulfinic acids and display characteristic redox reactivity in both synthetic and catalytic contexts. Selenoxides participate in elimination reactions, while seleninic acids act as oxidants and catalysts [96]. Other specialized organoselenium compounds include heterocyclic selenides such as ebselen, selenoureas, selenoketones, and selenocyanates, which exhibit diverse biological activities [108]. Additionally, cyclic and mixed chalcogen compounds, such as seleniranes and thioselenides, further expand the structural diversity and functional applications of organoselenium chemistry, with mixed Se–S systems and three-membered selenium heterocycles documented for their unique reactivity [109].

### 2.1. Seleninic Acids

Seleninic acids (Figure 3, Table 1) are organoselenium oxoacids analogous to sulfinic acids and constitute a stable class of selenium(IV) derivatives that are isolable and useful in synthetic chemistry. They are typically prepared through the oxidation of diselenides or suitable selenium precursors using oxidants such as hydrogen peroxide, nitric acid, permanganate, or dimethyldioxirane, or via hydrolysis of organoselenium trihalides and related intermediates, which allows generation of both aromatic and aliphatic seleninic acids in high purity and yield [96]. Seleninic acids are valuable oxygen-transfer agents, catalyzing a variety of oxidation reactions such as epoxidation of alkenes, Baeyer–Villiger oxidations, and peroxide-mediated substrate oxidations; mechanistic studies indicate that Se(IV) seleninic acid species can activate H_2_O_2_ and undergo interconversion with higher oxidation states under reaction conditions, thereby affecting catalytic efficiency and selectivity [110]. Despite their utility, limitations in synthesis and catalytic application arise from the need for strong oxidants and careful control of reaction conditions to avoid overoxidation to selenonic acids or decomposition pathways. Additionally, the stability and reactivity balance of seleninic acids can be sensitive to substituent effects and solvent environments, leading to competing pathways such as disproportionation or formation of side products when harsh conditions are employed [96]. From a synthetic perspective, the relatively straightforward oxidative preparation contrasts with the challenge of fine-tuning Se(IV)/Se(VI) redox cycles during catalysis, as evidenced by mechanistic work showing that Se(VI) “peroxyselenonic” intermediates may be more active oxidants in some transformations but may also be more prone to deactivation under certain conditions [110]. These attributes, including ease of access via oxidation and participation in oxygen transfer reactions, make seleninic acids useful reagents in organic synthesis, particularly where selective oxidation is desired. On the other hand, reaction design is required to mitigate overoxidation and decomposition.

### 2.2. Selenocysteine

SeCys (Figure 3, Table 1) is a selenium-containing amino acid characterized by a highly reactive selenol functional group. This moiety distinguishes SeCys from its sulfur analogue, cysteine, and accounts for its enhanced nucleophilicity and distinctive redox behavior in chemical systems, because selenium’s lower electronegativity and larger atomic radius compared to sulfur facilitate Se-C bond reactivity and redox transformation propensity [111]. Chemically, SeCys residues are typically introduced into peptides and small proteins via solid-phase peptide synthesis (SPPS) using protected SeCys building blocks, peptide ligation methods such as native chemical ligation (NCL), and controlled diselenide metathesis strategies, with careful protection of the selenol group required during chain assembly to minimize oxidation and side reactions [112]. The high reactivity of the selenol moiety presents a limitation in synthesis and handling, as the free selenol readily oxidizes to diselenide and other Se-derived species under aerobic conditions, necessitating inert atmosphere techniques or protective groups to preserve the desired SeCys functionality during multistep synthesis [112]. Additional constraints in the chemical use of SeCys include sensitivity to oxidative degradation, challenges in selective deprotection without side reactions, and difficulties in large-scale preparation of SeCys-containing peptides with uniform regiochemistry and stereochemical integrity [112].

### 2.3. Selenomethionine

SeMet (Figure 3, Table 1), similar to SeCys, is a naturally occurring selenium-containing amino acid in which selenium replaces sulfur in methionine, resulting in a chiral molecule at the α-carbon with physicochemical properties closely related to those of its sulfur analogue but with enhanced polarizability and redox responsiveness due to the presence of selenium [111]. Chemically, SeMet can be synthesized through substitution reactions starting from protected methionine derivatives or via selenium-containing alkylation strategies, while scale-up production is more commonly achieved through microbial or yeast-based biosynthetic enrichment systems that incorporate selenium into methionine analogues under controlled conditions [113]. SeMet is incorporated into proteins in place of methionine and used in protein crystallography due to selenium’s X-ray scattering properties [114].

From a structural and analytical perspective, SeMet is widely employed in protein crystallography as a methionine substitute, as the selenium atom provides strong anomalous X-ray scattering that facilitates phase determination in single-wavelength anomalous dispersion (SAD) and multi-wavelength anomalous dispersion (MAD) experiments [113]. Despite its relative stability compared to SeCys, limitations associated with SeMet include susceptibility to oxidative degradation under harsh conditions, potential non-specific substitution for methionine in synthetic and recombinant systems, and challenges in controlling selenium speciation during large-scale preparation and storage [115]. Additionally, the chemical similarity between sulfur- and selenium-containing methionine analogues can complicate selective reactivity and analytical discrimination in complex chemical or biochemical matrices [116].

### 2.4. Selenols

Selenols (Table 1, Figure 3) are the selenium analogs of thiols, characterized by a Se-H bond, as described in early organoselenium synthesis and characterization studies where selenols were first identified and their fundamental properties were established [96,117,118]. Owing to selenium’s larger atomic radius and lower electronegativity compared to sulfur, selenols are highly nucleophilic and significantly more reactive than their sulfur counterparts. This makes them particularly susceptible to oxidation, often forming diselenides or selenenic acids under physiological conditions, consistent with mechanistic studies on organoselenium intermediates that document the formation and instability of selenenic acid intermediates during the oxidation of selenols [20,96,119].

Selenols are structurally categorized into primary (R–CH_2_SeH), secondary (R_2_–CHSeH)**,** and tertiary (R_3_–CSeH) categories, mirroring the classification of alcohols. Primary selenols feature a SeH group on a carbon bonded to one organic group and two hydrogens. Secondary selenols have the SeH-bearing carbon attached to different organic substituents. Tertiary selenols, bonded to three organic groups, are typically achiral and suffer from significant steric hindrance, which compromises their stability; such steric and electronic influences on organoselenium compound stability and reactivity are discussed in comprehensive reviews of organoselenium chemistry that cover steric effects on Se–H reactivity and decomposition pathways [99,118].

The chemical behaviour of selenols, particularly their highly polarizable Se–H bond and redox sensitivity, makes them important intermediates in synthetic and catalytic organoselenium chemistry, where selenols, selenenyl sulfides, and related species participate in controlled oxidation and reduction transformations, as described in mechanistic reviews of organoselenium redox chemistry [20,99].

#### 2.4.1. Conventional Methods for the Synthesis of Selenols

Conventional synthesis of selenols typically involves nucleophilic substitution reactions, where a leaving group is displaced by a selenium-based nucleophile such as hydrogen selenide (HSe^⊝^) or selenolate anions (RSe^⊝^) [120].

**Scheme 1:**
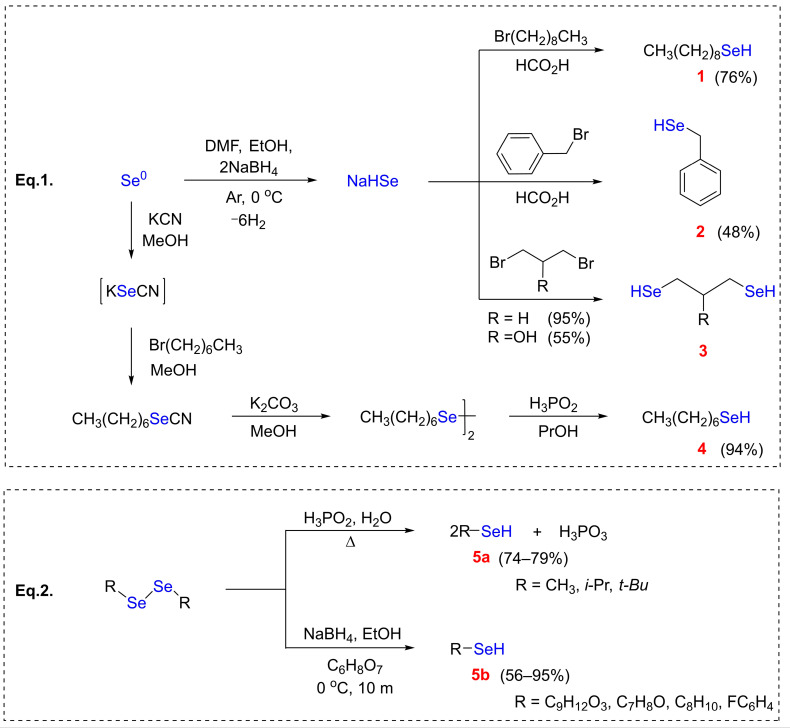
Conventional synthetic routes for selenols.

Krief and co-workers reported a facile one-pot technique for synthesizing aliphatic selenols from common alkyl halides, elemental selenium, and sodium borohydride [121]. In this reported approach, elemental selenium [Se(0)] is reduced in situ by sodium borohydride (2 equiv) in dimethylformamide/ethanol (6 equiv) at 0 °C under argon to generate complex A (sodium hydroselenide) (Scheme 1, *Eq.1*). Treatment of complex A with formic acid and alkyl halides afforded alkyl selenols **1** (76%) and **2** (48%) in satisfactory yields [121].

Similarly, reaction of sodium hydroselenide with 1,3-dibromopropane or 1,3-dibromo-2-propanol, followed by acidification with hydrogen chloride, furnished the corresponding diselenols **3** (R = H, 95%; R = OH, 55%) after extraction (Scheme 1, *Eq.1*) [122,123]. Notably, 1,3-propanediselenol was later employed as a key building block in the synthesis of linear and macrocyclic Ni-bridged Fe/Se cluster complexes [124]. Despite its experimental simplicity, this approach suffers from a major limitation because it involves the formation and handling of highly toxic hydrogen selenide during the acidification step.

To address safety and operational concerns, Blacker et al. developed a four-step telescoped protocol for the synthesis of 1-alkaneselenols **4** (94%) via selenocyanate intermediates [125]. In this method, potassium selenocyanate is generated in situ from potassium cyanide and elemental selenium, then reacted with alkyl halides to afford the corresponding alkyl selenocyanates (Scheme 1, *Eq.*1) [125]. These intermediates are converted into dialkyldiselenides, which are subsequently reduced using hypophosphorous acid to yield the desired 1-alkaneselenols **4**. Importantly, the first three transformations are carried out sequentially in methanol without isolation of intermediates, minimizing exposure to unstable and malodorous selenium species. The resulting diselenides may be distilled prior to reduction or reduced directly, with both approaches providing comparable yields and purities [125].

The research performed by Günther demonstrated that the reduction of diselenides with aqueous hypophosphorous acid proceeds with a 1:1 stoichiometry, producing two equivalents of selenol per diselenide [126]. Thermal treatment of dimethyl diselenide with 50% hypophosphorous acid resulted in vigorous gas formation and formation of volatile methylselenol **5a** (74–79%), which was isolated by cryogenic condensation (Scheme 1, *Eq.*2) [127]. More recently, Tanini and Capperucci reported an alternative and milder reduction protocol for diselenides using sodium borohydride, followed by protonation with citric acid (C_6_H_8_O_7_). This method enabled the efficient synthesis of a broad range of aryl selenols **5b** (56–95%) (Scheme 1, *Eq.*2). During optimization, citric acid proved superior to other inorganic and organic proton sources, affording high yields while maintaining operational simplicity [125,128].

#### 2.4.2. Green Syntheses of Selenols

**Scheme 2:**
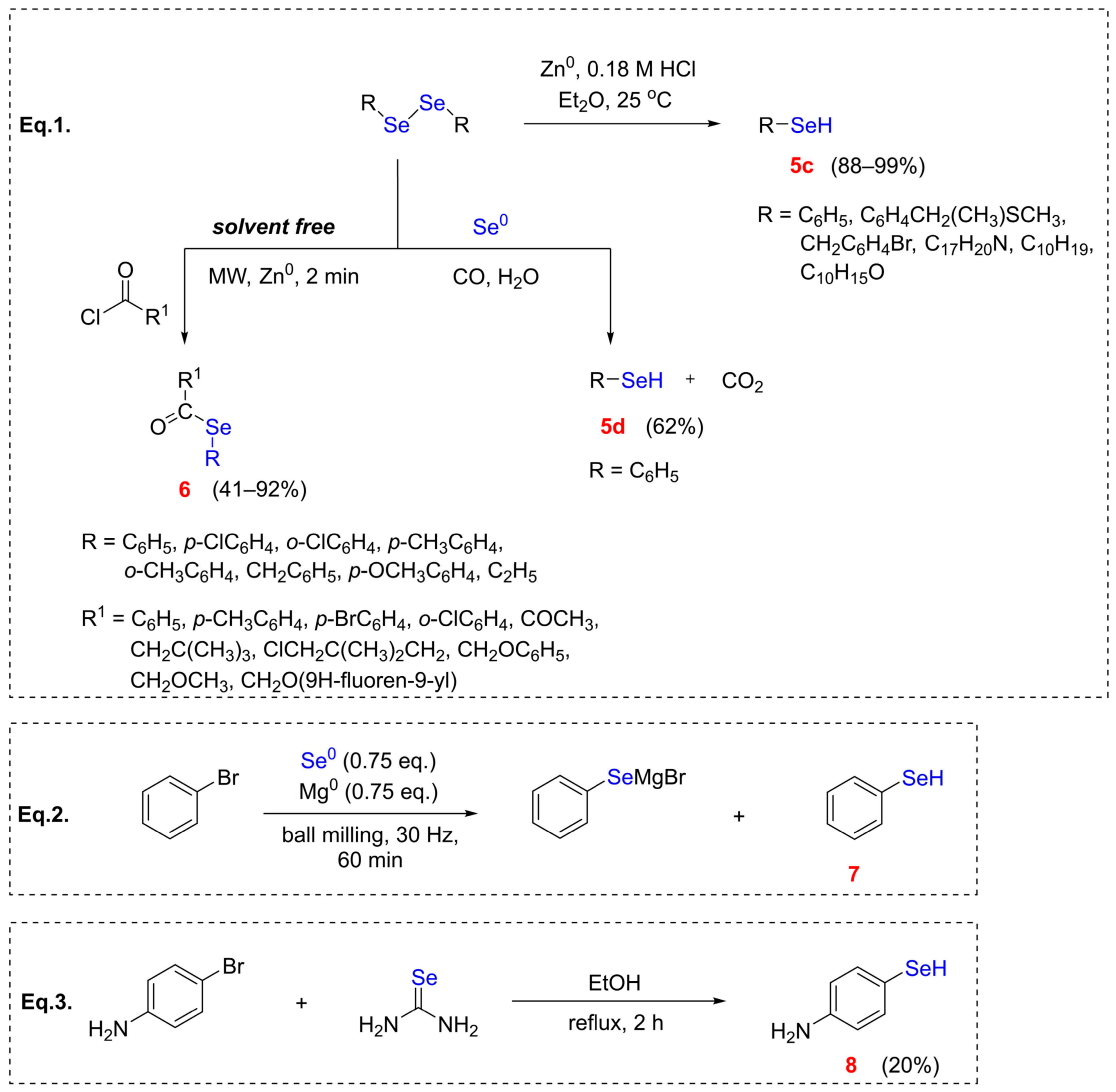
Green synthesis of the selenols.

Alternative reduction strategies have been explored for the conversion of diselenides into selenols. For instance, selenium, carbon monoxide, and H_2_O have been used as a reducing system to transform diphenyl diselenide into benzeneselenol. Among these methods, zinc-mediated reduction has emerged as an efficient and environmentally benign approach. Santi and co-workers developed a zinc-based protocol in which diselenides are reduced to the corresponding selenols **5c** (88–99%) in a biphasic aqueous/organic system (H_2_O/diethyl ether) under acidic conditions (Scheme 2, *Eq.1*) [129]. This methodology not only provides good yields of selenols but also offers several advantages from a green chemistry perspective. The use of zinc, an inexpensive and relatively non-toxic metal, along with H_2_O as part of the reaction medium, reduces the reliance on hazardous organic solvents and harsh reagents. Moreover, the biphasic system facilitates product isolation and minimizes waste, making the procedure more sustainable compared to conventional reduction methods.

Microwave irradiation has emerged as a powerful tool in organic synthesis, often providing higher product yields, suppressing the formation of side products, and enabling reactions to proceed under milder conditions with significantly shorter reaction times. From an environmental standpoint, the combination of microwaves with solvent-free conditions has been recognized as an eco-friendly alternative to conventional heating methods, as it reduces energy consumption and minimizes the use of hazardous solvents. Despite these advantages, the application of microwave technology in organoselenium chemistry remains limited, and reports exploring the synergistic use of microwaves and solvent-free conditions in this field are particularly scarce. Braga et al. have reported and developed a sustainable, environmentally benign approach to the synthesis of selenol esters. This method employs microwave irradiation under solvent-free conditions, enabling the rapid formation of selenol esters **6** (41–92%) within a very short reaction time of 2 min (Scheme 2, *Eq.1*) and highlighting both operational simplicity and adherence to green chemistry principles [130]. The selenol esters can subsequently be converted into the corresponding selenols through simple hydrolysis or reduction, demonstrating how this green and efficient approach directly contributes to the preparation of selenols.

In the course of studies aimed at investigating the reducing properties of in situ generated hydrogen selenide toward various functional groups, Sonoda et al. observed that diselenides can be readily reduced to the corresponding selenols. For example, diphenyl diselenide was efficiently reduced to benzeneselenol **5d** (62%) under the pressure of carbon monoxide at 70 °C, using an equimolar amount of selenium and *N*-methylpyrrolidine as a base [131]. The benzeneselenol formed was successfully isolated by distillation, demonstrating the effectiveness of this reduction method (Scheme 2, *Eq.1*) [131]. This method can be considered greener than traditional multi-step or harsh reduction protocols, as it offers a more direct and efficient route to selenols while operating under relatively mild conditions. However, the involvement of carbon monoxide as a reactant and the use of selenium still present environmental and safety concerns that must be carefully managed.

Following a mechanochemical study (Scheme 2, *Eq.2*), Chen et al. demonstrated that under an argon atmosphere, with work-up performed by short-column filtration inside a glove box, the reaction exclusively produced benzeneselenol **7** [132]. To confirm the formation of magnesium-based selenium nucleophiles under ball-milling conditions, their research employed near-edge X-ray absorption fine structure (NEXAFS) spectroscopy. The mechanochemically generated selenium nucleophile was transferred under argon into the soft X-ray optics for analysis. The NEXAFS measurements revealed a high-energy shift in the Mg K-edge absorption (1317.0 eV) relative to the Mg^0^ edge of a standard magnesium metal flake (1315.0 eV), unambiguously confirming the presence of divalent Mg^2+^ species in the solid-state mixture. These results indicate that the mechanochemical ball-milling of magnesium metal with elemental selenium generates in situ reactive magnesium–selenium nucleophiles, which under inert argon conditions can be directly converted to benzeneselenol. This approach minimizes side reactions and enables the selective formation of the selenol. The NMR of the crude mixture reported only ~5% benzeneselenol [132]. An alternative approach to the synthesis of 4-aminobenzeneselenol **8** (20%) was reported by Ruberte et al., in which a 1:1.1 molar mixture of 4-bromoaniline and selenourea was heated under reflux in ethanol. Under these conditions, nucleophilic selenium generated in situ from selenourea displaced the aryl bromide, leading to formation of the desired selenol, which was obtained in moderate yield (Scheme 2, *Eq.3*) [133].

### 2.5. Ebselen

Ebselen (Figure 3, Table 1) is a synthetic organoselenium compound classified as a benzisoselenazolone, featuring a five-membered heterocyclic ring containing both selenium and nitrogen atoms [134]. Unlike classical organoselenium compounds such as selenols or selenides, ebselen contains a Se-N bond within a rigid aromatic framework, which defines its unique structural and mechanistic characteristics. The core benzisoselenazolone scaffold facilitates redox cycling and reversible thiol interactions, thereby chemically mimicking the catalytic behavior of selenoenzymes [118]. Its selenium center undergoes redox transitions between Se(II) and Se(IV) oxidation states via the formation of selenol and selenenic acid intermediates during catalytic cycles. These transformations allow ebselen to act as a synthetic redox mediator, and its reactivity can be exploited in thiol-mediated catalysis, selenium transfer reactions, and organoselenium synthesis [98,135,136,137]. Mechanistically, ebselen reacts with small-molecule thiols such as glutathione (GSH) or other nucleophilic species, forming Se–S intermediates that can be reduced to regenerate the active compound, thus enabling controlled redox cycling [138].

The absence of a free selenol group in ebselen renders its selenium center less prone to nonspecific oxidation, providing a more predictable reactivity profile compared with many other organoselenium compounds. This chemical stability, combined with its Se-N heterocyclic framework, makes ebselen a versatile scaffold for the design of synthetic organoselenium derivatives, redox-active catalysts, and selenium-containing heterocycles [139,140].

#### 2.5.1. Conventional Methods for the Synthesis of Ebselen and Its Derivatives

**Scheme 3:**
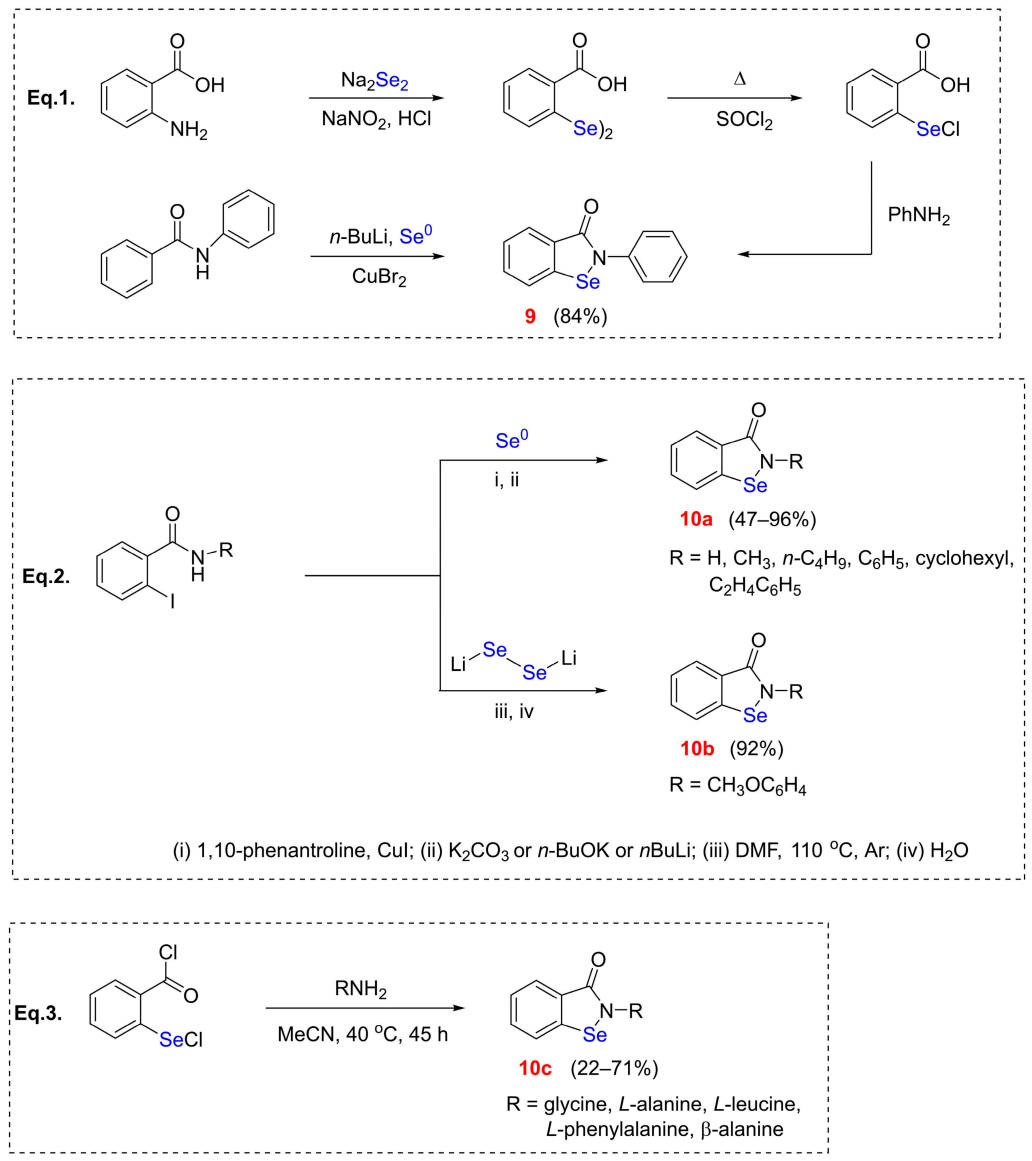
Conventional synthesis of ebselen and its derivatives.

The synthesis of ebselen **9** (84%; Scheme 3, *Eq.1*) remains challenging and currently relies primarily on two methods as described [141]. One method involves the ortho-lithiation of benzanilide, while the other employs a multistep synthetic route based on bis(ortho-benzoic acid) diselenide. Both strategies present practical and methodological limitations, underscoring the need for more efficient and broadly applicable procedures for constructing the selenazolone framework [141].

Balkrishna et al. reported an efficient catalytic method for synthesizing biologically relevant Se-N heterocycles **10a** (47–96%; Scheme 3, *Eq.2*) from halo-amides and elemental selenium in the presence of a base [141,142,143,144]. Pacula et al. demonstrated that the reaction of *o*-iodobenzamides with Li_2_Se_2_ can be selectively controlled by the presence of H_2_O, offering a simple and efficient protocol for the synthesis of benzisoselenazolones. Using this approach, a series of *N*-aryl ebselen **10b** (92%; Scheme 3, *Eq.2*) derivatives were successfully prepared [143].

In the original work by Młochowski et al., 2-(chloroseleno)benzoyl chloride was reacted with various amino acids or their esters under mild conditions to afford 2-carboxyalkyl-1,2-benzisoselenazol-3(2H)-ones [145]. This transformation proceeds through a tandem nucleophilic acyl substitution and intramolecular cyclization, exploiting the bifunctional electrophilicity of the selenium-activated benzoyl chloride moiety. The amino group of the amino acid attacks the acyl chloride carbonyl, forming an amide intermediate, which then undergoes intramolecular cyclization via Se-N bond formation to generate the benzisoselenazolone ring system. The reactions are typically performed in acetonitrile under controlled temperature conditions, where the polar aprotic solvent facilitates nucleophilic attack by the amino group while minimizing competing hydrolysis of the acyl chloride. Notably, the reaction proceeds smoothly without additional activating agents, highlighting the intrinsic reactivity of the chloroseleno benzoyl chloride. Upon completion, the amide intermediate cyclizes efficiently to yield the corresponding *N*-substituted benzisoselenazol-3(2H)-one, bearing a carboxyalkyl substituent at the nitrogen atom [145].

Extending this methodology, Pacuła et al. introduced a revised synthetic protocol to improve reaction efficiency, reproducibility, and compatibility with chiral amino acids. Using this optimized approach, a series of N-substituted benzisoselenazol-3(2H)-ones **10c** (22–71%; Scheme 3, *Eq.3*) derived from glycine, L-alanine, L-leucine, L-phenylalanine, and β-alanine were successfully synthesized [142,145]. Importantly, the revised procedure preserved the stereochemical integrity of chiral amino acids such as L-alanine, L-leucine, and L-phenylalanine, affording optically pure products without detectable racemization. This feature is crucial for biological evaluation, as the stereochemistry of the amino acid side chain significantly influences biological activity. In the case of β-alanine, the increased flexibility of the linker resulted in a structurally distinct N-substituted derivative, demonstrating the versatility of the method toward both α- and β-amino acids.

#### 2.5.2. Green Synthesis Approaches for Ebselen Derivatives

**Scheme 4:**
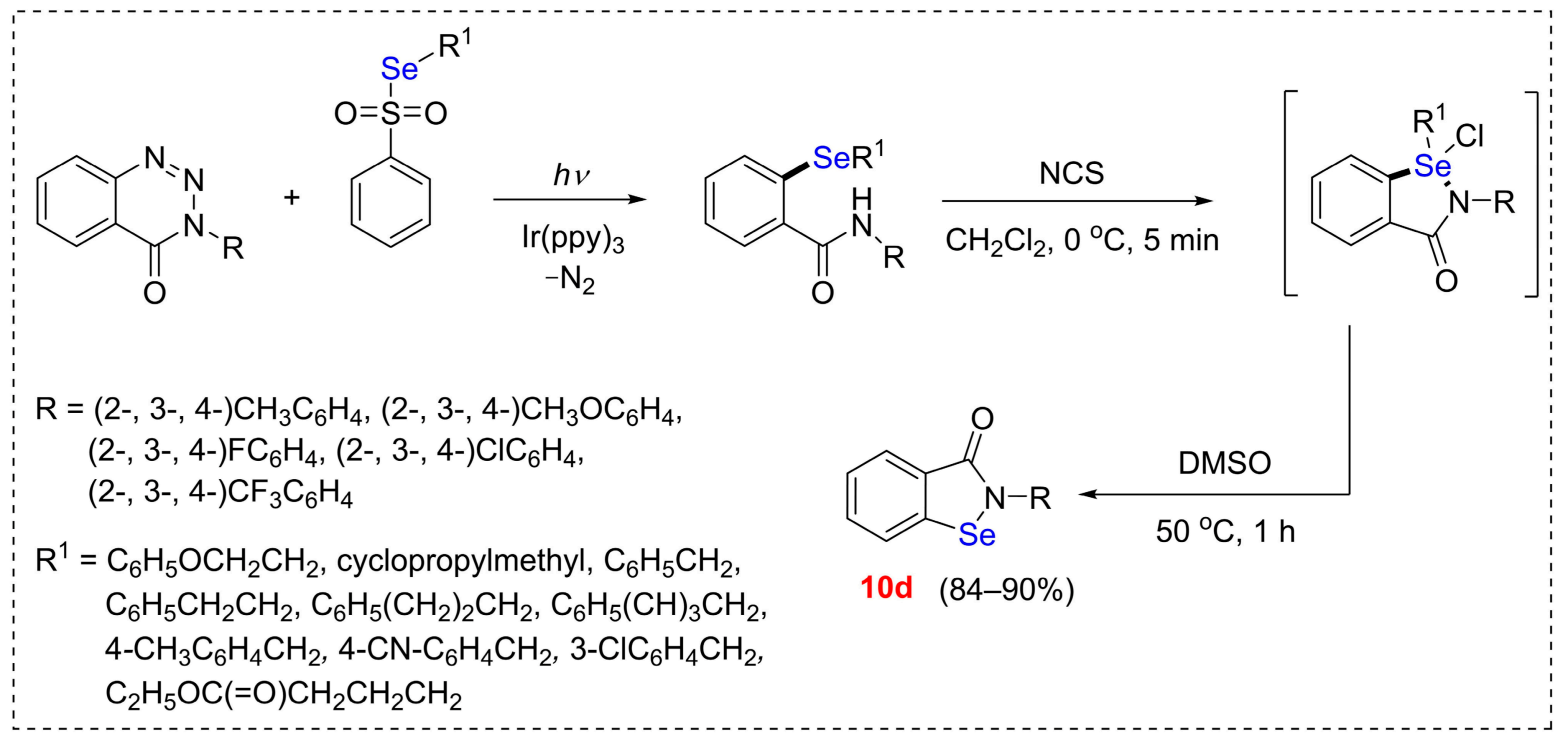
Green synthesis of ebselen derivatives.

Liu et al. reported an Ir-(ppy)_3_-hν-promoted denitrogenative *ortho*-selenylation (Scheme 4), a visible-light-mediated photocatalytic method for selectively introducing selenium at the ortho position of benzotriazinone derivatives [146]. In this transformation, the iridium(III) photocatalyst fac-Ir(ppy)_3_ absorbs visible light and enters an excited state, initiating single-electron transfer that activates the benzotriazinone substrate. The substrate undergoes denitrogenation, releasing N_2_ and generating a reactive intermediate at the *ortho* position, which then couples with a selenium donor such as a selenosulfonate to form the C-Se bond.

In some variants, *N*-chlorosuccinimide (NCS) is employed as a mild oxidant to facilitate selenium incorporation or improve reaction efficiency. The reaction proceeds under mild room-temperature conditions, avoids harsh thermal or stoichiometric reagents, and provides regioselective *ortho*-selenylation, making it an efficient and environmentally favorable strategy for constructing selenium-containing heterocycles **10d** (84–90%; Scheme 4) [146].

### 2.6. Selenides and Diselenides

Selenides (Figure 3, Table 1) are selenium analogs of ethers, consisting of a selenium atom bonded to two organic substituents [147]. These compounds play a central role in redox chemistry and have been widely employed as ligands in coordination chemistry, as selenium transfer agents, and as precursors to selenium-based redox catalysts [20]. Under mild oxidation conditions, selenides are converted to selenoxides, which can undergo *syn*-elimination reactions, while more oxidative environments can yield diselenides through oxidative coupling [20]. The inherent reactivity of selenides has also been exploited in the synthesis of more complex organoselenium frameworks, including selenoesters, selenoamides, and benzisoselenazolones [148].

Diselenides (Figure 3, Table 1) are structural analogs of disulfides and are characterized by a Se–Se bond. They serve as key intermediates in the oxidation and reduction of selenols and readily participate in reversible redox reactions, making them valuable in the development of synthetic organoselenium systems [90,149]. Diselenides have been extensively used as synthetic building blocks, with their Se-Se bond cleavage enabling the preparation of selenol-containing compounds, selenium heterocycles, and various functionalized organoselenium derivatives [150].

#### 2.6.1. Conventional Methods for the Synthesis of Selenides

**Scheme 5:**
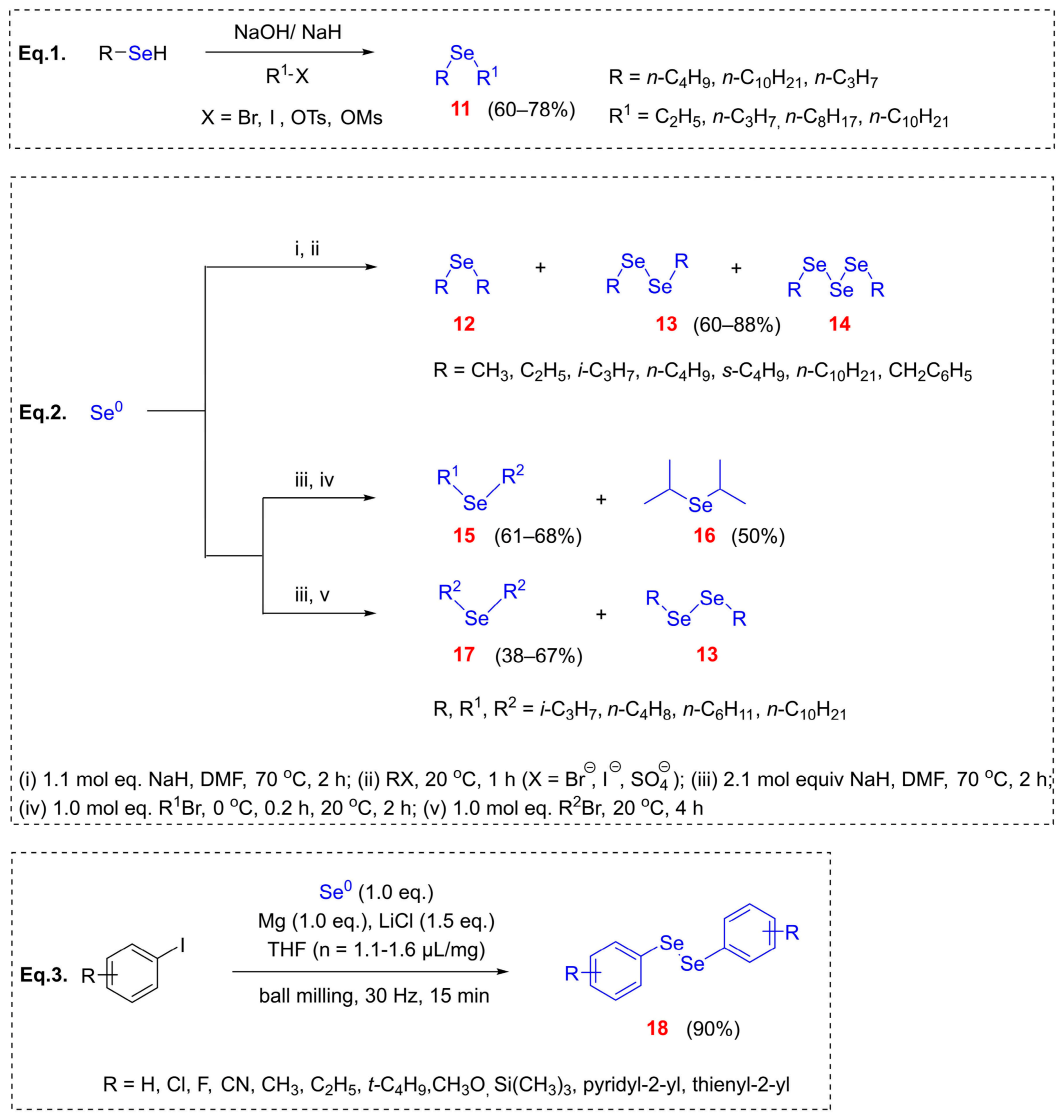
Conventional synthesis of selenides and diselenides.

In the approach shown in Scheme 5, *Eq.1*, selenols are first converted into their corresponding selenolate species by treatment with an excess of base, such as sodium hydroxide or sodium hydride. As reported by Tanini and Capperucci, the in situ produced selenolates act as highly nucleophilic intermediates that efficiently undergo alkylation with a variety of alkyl halides, furnishing unsymmetrical dialkyl selenides **11** (60–78%; Scheme 5, *Eq.1*) [122]. This sequence represents a practical and operationally straightforward method for C–Se bond formation, displaying broad functional group tolerance and compatibility with sensitive substituents. The mild reaction conditions, combined with the versatility of both selenol and halide partners, make this approach a valuable strategy for the synthesis of structurally diverse organoselenium compounds [122].

Elemental selenium is a practical selenium precursor in modern organoselenium chemistry; its availability and stability make it a versatile and cost-effective source for the synthesis of selenides and diselenides, enabling direct C–Se bond formation under relatively mild conditions [151,152]. In this context, reductive activation of elemental selenium provides reactive selenium species that, upon alkylation, preferentially afford diselenides.

Sodium hydride has been shown to be an efficient reducing agent for the in situ generation of sodium diselenide from elemental gray selenium in dimethylformamide [153]. Notably, dimethylformamide also serves as an excellent solvent for the subsequent alkylation step. Optimal reaction conditions involve heating the mixture at 70 °C for 2 h using 1.1 equivalents of sodium hydride per equivalent of selenium. The resulting sodium diselenide is then alkylated at room temperature with one equivalent of the appropriate alkyl halide. Under these conditions, dialkyl diselenides **13** (60–88%; Scheme 5, *Eq.2*) are obtained as the major products, with only trace amounts of the corresponding dialkyl selenides **12** and dialkyl triselenides **14** (Scheme 5, *Eq.2*). These minor byproducts can be readily removed by distillation or silica gel column chromatography. Although the reduction of elemental selenium can be conducted at lower temperatures, such conditions lead to diminished selectivity and increased formation of dialkyl selenides **12** and triselenides **14**, at the expense of the desired dialkyl diselenides **13**, as reported by Krief and Derock [153].

The synthesis of unsymmetrical dialkyl selenide **15** (61–68%; Scheme 5, *Eq.*2) was carried out in dimethylformamide, which afforded excellent selectivity, although the overall yields remained moderate. In contrast, reactions performed in *N*-methyl-2-pyrrolidone resulted in poor selectivity and low yields. Notably, the formation of di-isopropyl selenide **16** (50%; Scheme 5, *Eq.*2) was observed as a minor byproduct under the following conditions, routes iii and iv. In this procedure, elemental selenium is first treated with 2.1 mol equivalents of sodium hydride in dimethylformamide at 70 °C for 2 h to generate sodium diselenide in situ. The resulting reactive species is then alkylated at room temperature over 2 h with 1 mol equivalent of the desired alkyl bromide (R^1^Br), efficiently producing the corresponding dialkyl diselenide. This two-step, one-pot approach allows for controlled formation of the C–Se bond under mild conditions, while minimizing byproduct formation and providing good functional group tolerance [153]. On the other hand, under the following routes iii and v (2.1 mol equiv. sodium hydride, dimethylformamide, 70 °C, 2 h; then 1 equiv. R^2^Br, 20 °C, 4 h), dialkyl selenide **17** (38–67%; Scheme 5, *Eq.2*) formed alongside substantial dialkyl diselenide **13** (Scheme 5, *Eq.2*), indicating that sodium selenide likely acts as the reactive intermediate.

Following Scheme 5, *Eq.3*, a series of iodobenzene derivatives was employed as a model substrate in a mechanochemical reaction with magnesium, elemental selenium, and lithium chloride in tetrahydrofuran under stainless steel milling conditions [132]. The reaction proceeded rapidly and efficiently, with complete consumption of the starting material within 15 min. Interestingly, instead of the anticipated benzeneselenol, the transformation produced diphenyl diselenide **18** (90%; Scheme 5, *Eq.3*) in high yield [132]. This result underscores the effectiveness of mechanochemical activation for selenium, offering a solvent-minimized, rapid, and high-yielding approach to symmetrical diselenides. By avoiding the formation of benzeneselenol as the major product and utilizing elemental selenium directly, this method represents a practical and sustainable alternative to conventional solution-phase synthesis. The findings suggest that mechanochemistry can be further exploited to broaden the scope of selenium-containing compounds, providing an operationally simple and environmentally friendly route for organoselenium synthesis.

The use of tetrahydrofuran in ball-milling reactions raises concerns due to its toxicity, flammability, and environmental impact; therefore, such processes are often still classified as conventional rather than fully green, solvent-free or catalytic approaches. These results underscore the potential of mechanochemistry to expand the scope of organoselenium synthesis in a more operationally simple and environmentally conscious manner [154].

#### 2.6.2. Green Synthesis Methods for Selenides

In recent years, the development of environmentally benign methods for diaryl selenide synthesis, including solvent-free approaches and the use of alternative solvents, has gained attention. ILs, particularly 1,3-dialkylimidazolium-based salts, offer an attractive green solvent choice due to their negligible vapor pressure, nonflammability, thermal stability, ease of handling, recyclability, and potential for rate or selectivity improvements [155]. ILs have been successfully applied in various organic reactions, including palladium-catalyzed transformations, olefin metathesis, and asymmetric synthesis, often enhancing reaction rates compared to conventional solvents [156].

**Scheme 6:**
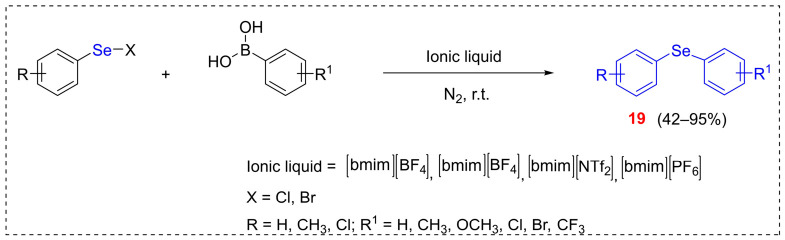
Green synthesis of selenides.

In organochalcogen chemistry, ILs have enabled the synthesis of vinylic selenides from phenylselenyl chloride and vinylboronic acids or esters with good yields and stereoselectivity [157]. Freitas et al. reported the synthesis of diaryl selenides **19** (42–95%; Scheme 6) via electrophilic substitution of arylboron reagents with arylselenium halides (X = Cl or Br) using imidazolium-based ionic liquids, [bmim]BF_4_, [bmim]NTf_2_, and [bmim]PF_6_, as solvents [157].

### 2.7. Selenoxides and Selenones

Selenoxides (Figure 3, Table 1) feature a selenium–oxygen double bond with tetrahedral geometry. They are particularly well-known for undergoing selenoxide eliminations, a powerful synthetic reaction widely employed in organic chemistry for the generation of alkenes. The relative stability of many selenoxides allows for isolation, structural characterization, and handling under controlled conditions [158]. Selenoxides are typically formed via oxidation of selenides using mild oxidants, and their reactivity can be exploited in synthetic sequences such as stereoselective eliminations, oxidation cascades, and selenium-mediated rearrangements [20,159]. Additionally, selenoxides can engage in thiol-exchange reactions and other nucleophilic transformations, providing a versatile platform for the construction of functionalized organoselenium compounds [90,160].

Selenones (Figure 3, Table 1) are the selenium analogs of sulfones, featuring a selenium atom doubly bonded to two oxygen atoms and substituted by dialkyl or diaryl groups [160]. These species are highly oxidized and chemically inert. However, their electrophilic selenium center enables selective reactions with nucleophiles, making them useful intermediates in synthetic organoselenium chemistry [161,162]. The planar geometry of selenones facilitates predictable reactivity in oxidation, substitution, and cyclization reactions. Furthermore, their oxidative stability and structural rigidity render them valuable as intermediates in the preparation of complex organoselenium frameworks, including selenazoles, benzisoselenazolones, and other heterocyclic scaffolds [71,163]. Furthermore, structural and electronic factors, such as significant steric hindrance or specific electronic configurations, can increase the activation energy and prevent the reaction from proceeding under typically mild conditions [164]. Selenones can also participate in electrophilic selenium transfer and other functionalization strategies, providing a chemically robust platform for developing diverse organoselenium derivatives [104,148].

#### 2.7.1. Conventional Synthesis of Selenoxides and Selenones

**Scheme 7:**
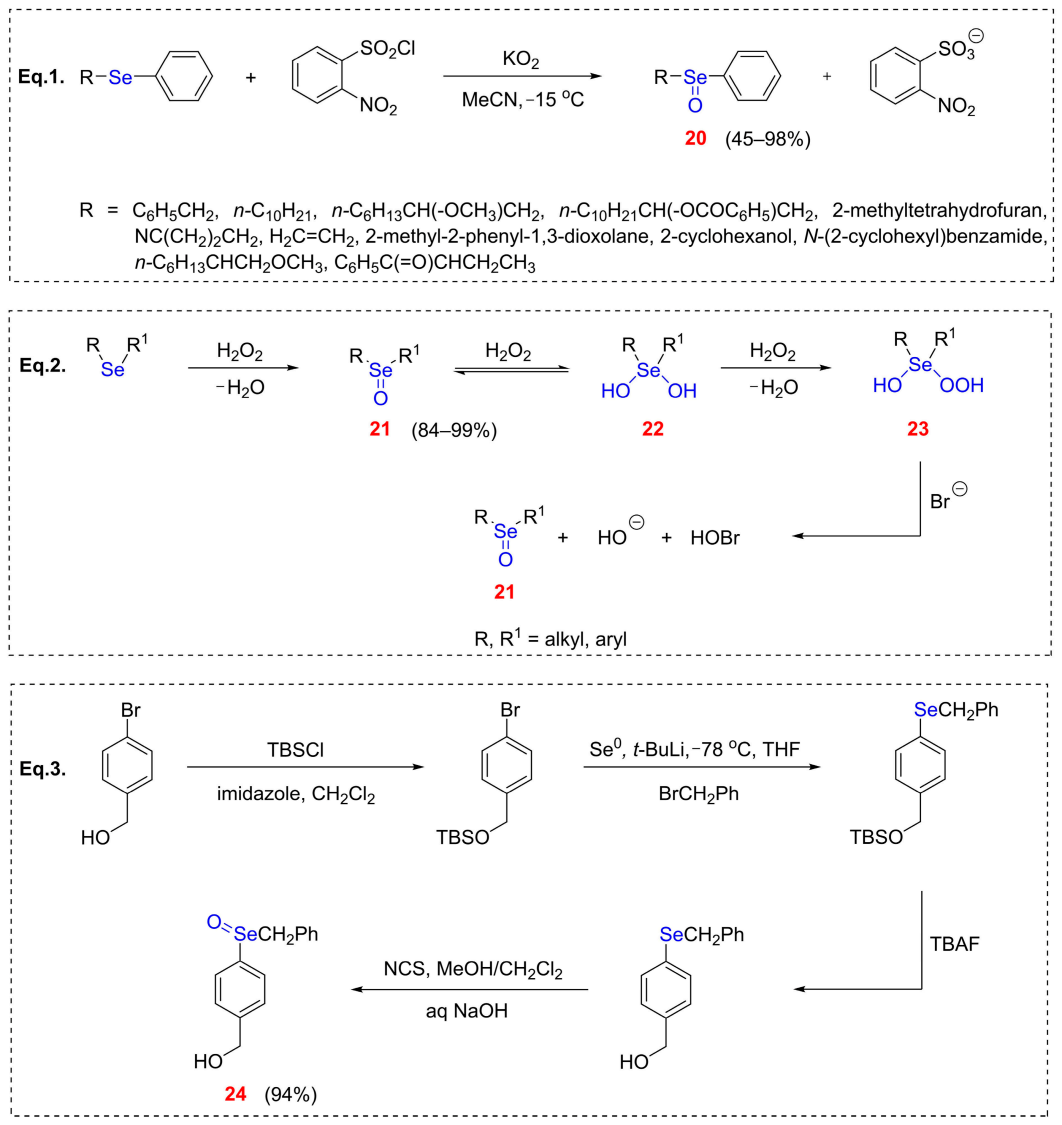
Conventional synthesis of selenoxides and selenones.

Tiecco et al. reported that various alkyl phenyl selenides can be oxidized to the corresponding selenoxides **20** (45–98%; Scheme 7, *Eq.1*) in excellent yields under mild conditions using 2-nitrobenzenesulfonyl peroxy intermediates [165]. These intermediates are generated in situ from the reaction of 2-nitrobenzenesulfonyl chloride with potassium superoxide at −15 °C in dry acetonitrile. The method, previously applied to the oxidation of sulfides to sulfoxides and sulfoxides to sulfones, relies on peroxy radical intermediates formed from the reaction of superoxide radical anions with sulfinyl, sulfonyl, or silyl chlorides, which act as more efficient oxidizing agents than superoxide itself [165].

Following Scheme 7, *Eq.2*, selenoxides **21** (84–99%) have emerged as highly efficient catalysts for a variety of oxidation reactions with hydrogen peroxide, including the bromination of organic substrates in the presence of sodium bromide. Upon reaction with H_2_O_2_, organic monoselenides are oxidized to selenoxides, whereas diselenides are oxidized to selenone **22** (Scheme 7, *Eq.*2) intermediates [166]. This process necessarily involves cleavage of the selenium–selenium bond. After oxidation, the resulting selenoxide functions as a peroxide activator and reacts in situ with H_2_O_2_ to form hydroxyperhydroxy selenane or peroxyseleninic acid **23** (Scheme 7, *Eq.*2), respectively.

These oxygen-transfer species can subsequently oxidize organic substrates [166]. These intermediates facilitate the oxidation of bromide ions to reactive brominating species that subsequently react with organic substrates to afford brominated products. The catalytic cycle is highly efficient because the selenoxide **21** is regenerated after each oxidation and allows for low catalyst loadings. Moreover, this reaction pathway offers significant environmental advantages because hydrogen peroxide is reduced to H_2_O and the organoselenium catalyst can often be recovered or immobilized for reuse. The high reactivity, versatility and recyclability of selenoxide catalysts make them valuable tools for selective and green oxidation methodologies [167].

A selenoxide bearing a benzyl alcohol functionality was synthesized as shown in Scheme 7, *Eq.3* [166]. The synthesis began with protection of 4-bromobenzyl alcohol as its *tert*-butyldimethylsilyl ether to prevent unwanted side reactions during lithiation. Treatment of the obtained intermediate with 2.2 equiv of *tert*-butyllithium generated a carbanion, which was reacted with elemental selenium and benzyl bromide to form selenide, introducing the selenium atom and the benzyl group. The TBS protecting group was then removed with tetrabutylammonium fluoride to yield selenide, and subsequent oxidation with NCS in methanol/dichloromethane, followed by aqueous sodium hydroxide hydrolysis, furnished the target selenoxide **24** (94%; Scheme 7, *Eq.*3) [166]. This sequence illustrates the strategic use of protection, lithiation, and controlled oxidation to access a functionalized selenoxide in a stepwise manner.

#### 2.7.2. Green Synthesis of Selenoxides and Selenones

Since the late 1970s, organoselenium compounds have been recognized as efficient catalysts for the activation of hydrogen peroxide, offering high chemo- and regioselectivity in diverse organic transformations [168]. These organoselenides serve as environmentally benign alternatives to traditional metal-based catalysts and have been successfully applied in a range of oxidative processes, including Baeyer–Villiger oxidations of ketones and aldehydes, epoxidation of alkenes, and the selective oxidation of alcohols and nitrogen-containing substrates [169,170].

**Scheme 8:**
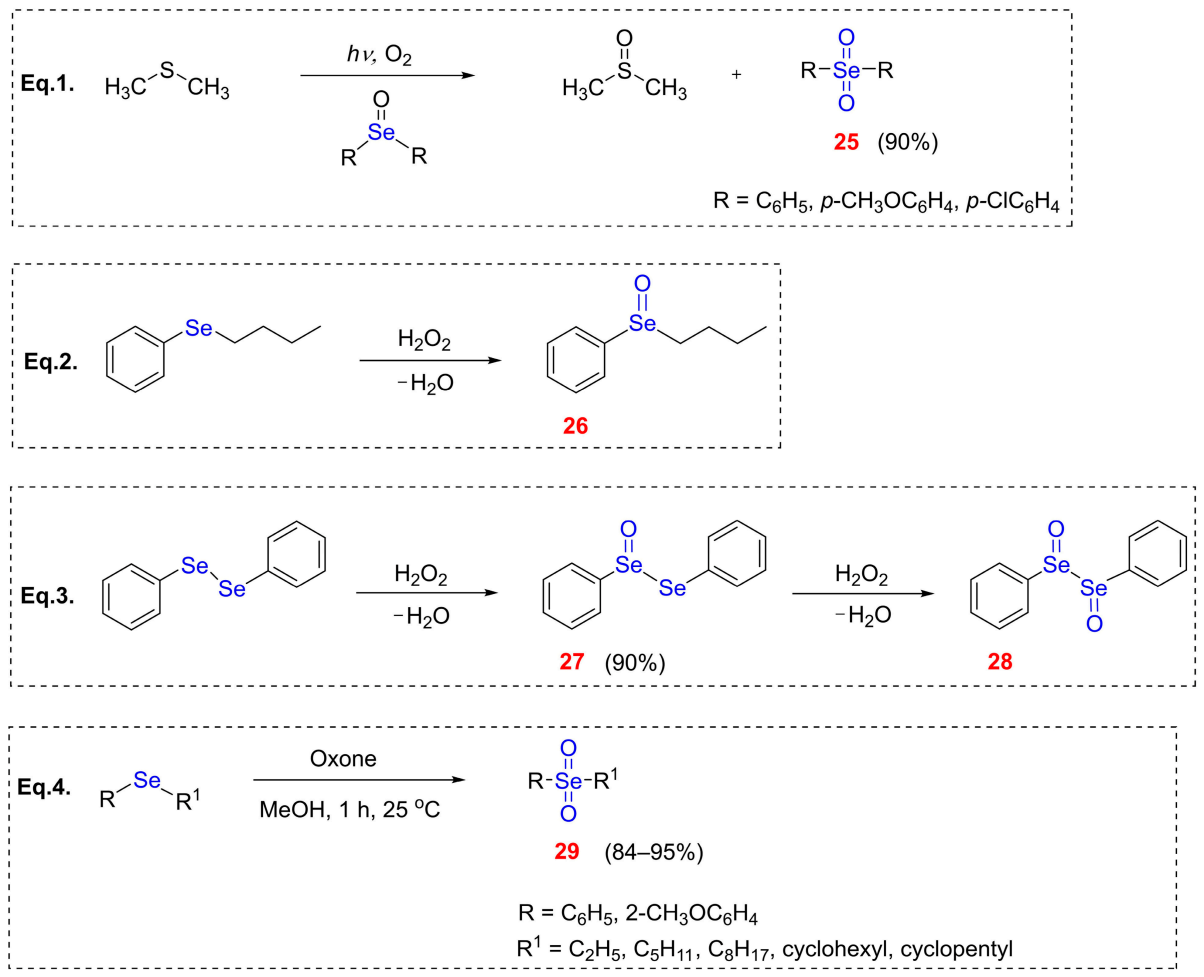
Green synthesis of selenoxides and selenones.

The reactivity of organoselenium compounds is closely tied to their ability to access higher oxidation states, a feature that is well illustrated by the stepwise oxidation of selenides to selenoxides and, upon further oxygen transfer, to selenones **25** (90%; Scheme 8, *Eq.1*) [171]. While the initial oxidation to the selenoxide is generally facile, the subsequent formation of selenium(VI) selenones introduces a markedly more electrophilic species, capable of engaging in a broader range of transformations. Within this oxidative continuum, the electronic nature of aryl substituents exerts a decisive influence on selenoxide behavior [171].

Systematic kinetic studies using diphenyl selenoxide **26** (Scheme 8, *Eq.2*) as a benchmark have shown that aryl substitution significantly modulates reactivity. The oxidation of phenyl-*n*-butyl selenide by hydrogen peroxide proceeds through direct nucleophilic attack of the selenium atom on the electrophilic oxygen of hydrogen peroxide, leading to formation of the corresponding selenoxide **26** (Scheme 8, *Eq.2*). During this process, one hydrogen from H_2_O_2_ is transferred to the selenium-bound oxygen, generating H_2_O as a byproduct [167]. Compound **26** should be regarded as a reactive intermediate rather than a fully stable and isolable molecule. Such oxidized organoselenium species are known to exhibit limited thermodynamic stability while remaining sufficiently persistent under the reaction conditions to participate in subsequent mechanistic steps. Therefore, the transient nature of compound **26** is consistent with its proposed role in the reaction pathway.

Ribaudo et al. described the stepwise oxidation of diaryl diselenides by hydrogen peroxide, providing a clear illustration of how substituent effects modulate selenium reactivity [167]. Oxidation of the parent diselenide initially affords the corresponding selenoxide **27** (90%; Scheme 8, *Eq.3*) with loss of water, while further oxidation under similar conditions leads to the formation of the higher-oxidation-state selenone **28** (Scheme 8, *Eq.3*). The efficiency and selectivity of these transformations are strongly influenced by the electronic nature of the aryl substituents, which govern both the reactivity of the intermediate selenoxide and its propensity to undergo further oxidation [167].

A particularly practical illustration of controlled selenone formation was provided by Ceccherelli and co-workers, who demonstrated that potassium hydrogen persulfate (oxone) serves as an efficient and mild oxidant for the stepwise oxidation of selenides to selenones **29** (84–95%), as illustrated in Scheme 8, *Eq.4* [17]. In this transformation, oxidation proceeds through initial formation of the corresponding selenoxides, followed by a second oxygen transfer to deliver selenium(VI) selenones. The reaction is notably sensitive to the reaction medium: optimal selenone formation was achieved using an excess of oxone in a methanol/H_2_O-buffered system at slightly basic pH, conditions that stabilize the oxidant and mitigate acid accumulation generated during oxidation. Under these carefully controlled conditions, a range of selenides was smoothly converted to the corresponding selenones in consistently high yields. This study underscores the importance of pH control and reaction environment in enabling efficient access to high-valent selenium species and highlights Oxone as a reliable reagent for selenone synthesis in preparative organoselenium chemistry [17].

### 2.8. Selenoureas

Selenoureas (Figure 3, Table 1) are planar analogs of ureas in which the carbonyl oxygen is replaced by selenium. Selenoureas can act as ligands for metal centers, forming stable metal–selenium complexes, and can participate in hydrogen-bonding interactions, which have been exploited in organocatalysis and materials chemistry [163,172].

The selenium atom in selenoureas is a versatile reactive site: it can participate in nucleophilic or electrophilic transformations, generate reactive selenium species (R-Se^⊝^, Se^0^), and serve as a precursor for the synthesis of selenazoles, benzisoselenazolones, selenolates, and other organoselenium derivatives [118,173]. Their ability to coordinate metals and undergo selenium transfer reactions makes selenoureas valuable intermediates in the design and preparation of complex organoselenium frameworks, including heterocyclic scaffolds and multifunctional selenium reagents.

#### 2.8.1. Conventional Synthesis of Selenoureas

In 1937, Douglass first reported the synthesis of acylselenoureas, which are typically generated in situ via the reaction of acid chlorides with potassium selenocyanate in acetone, leading to intermediate acyl isoselenocyanates along with other oligomeric species [174]. Owing to their limited stability, these intermediates are generally not isolated but are instead immediately reacted with primary or secondary amines to afford the corresponding acylselenoureas. However, the study was limited in scope, encompassing only a small number of products and lacking characterization using modern analytical techniques (^13^C and ^77^Se NMR and X-ray diffraction).

**Scheme 9:**
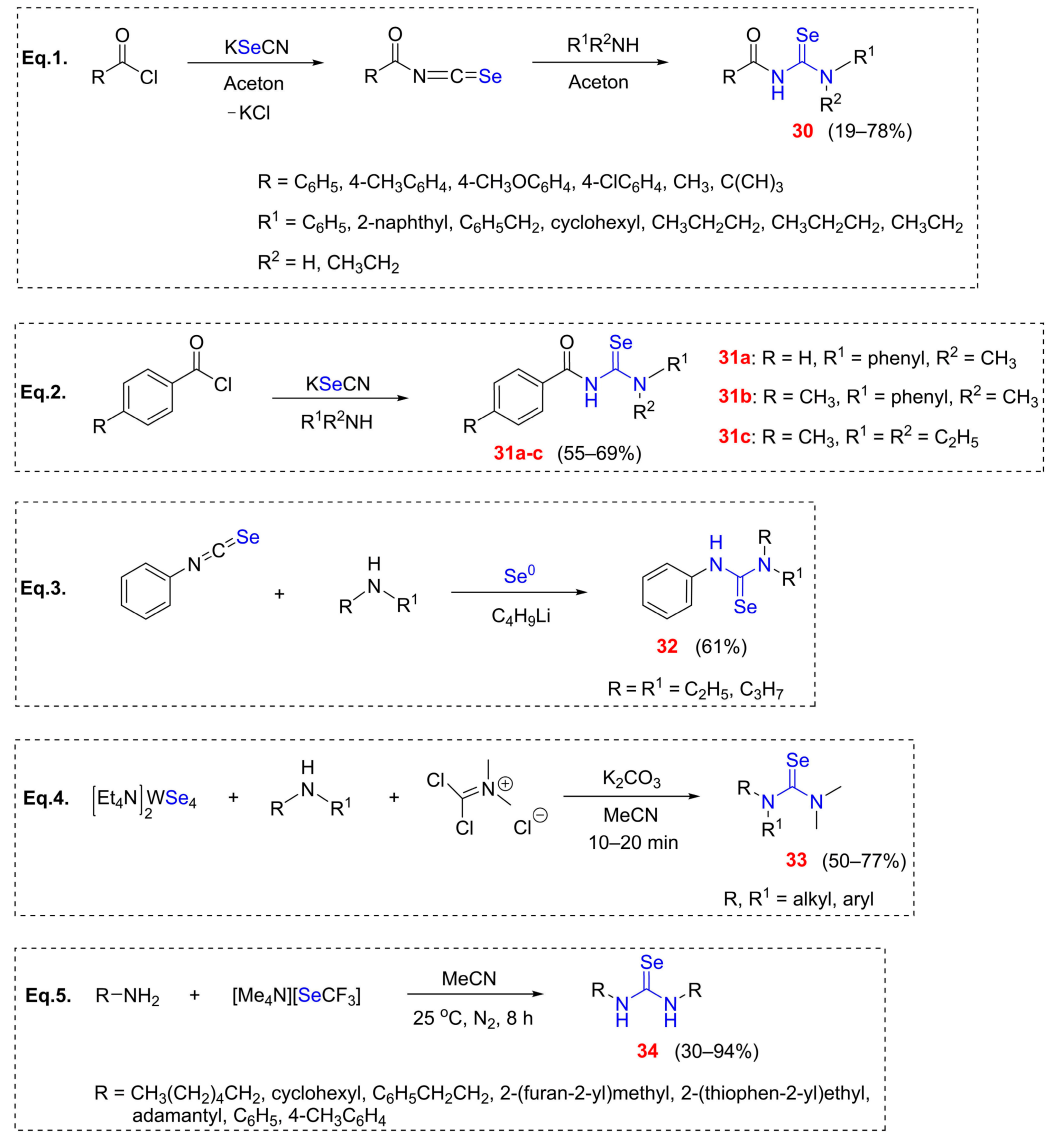
Conventional synthesis of the selenoureas.

Koketsu et al. in 2006 synthesized *N*-acylselenoureas via in situ formation of *p*-toluoyl isoselenocyanate, following the procedure reported by Douglass [175]. This methodology was extended to a variety of acyl chlorides and amines, affording a range of *N*-acylselenoureas **30** (19–78%) in moderate to high yields (Scheme 9, *Eq.1*) [175]. The protocol is compatible with different aromatic and aliphatic acyl groups, as well as primary and secondary amines, demonstrating broad substrate scope and functional group tolerance. All *N*-acylselenoureas were further characterized by NMR spectroscopy (^13^C and ^77^Se NMR) [175].

Molter et al. reported the synthesis of selenoureas **31a–c** using an efficient one-pot protocol, as illustrated in Scheme 9, *Eq.2* [176]. In this methodology, the corresponding aromatic acid chlorides were first reacted with potassium selenocyanate, followed by the subsequent addition of *N*-methylalanine or diethylamine, to generate acyl selenocyanate intermediates in situ. Subsequent addition of *N*-methylaniline or diethylamine enabled nucleophilic substitution at the selenium center, leading smoothly to the desired selenourea derivatives **31a–c** (55–69%; Scheme 9, *Eq.2*). This one-pot approach eliminates the need for isolation of reactive intermediates and proceeds under mild conditions, providing a practical and operationally simple route to structurally diverse selenoureas. The strategy highlights the utility of potassium selenocyanate as a convenient selenium source and demonstrates its effectiveness for the rapid construction of functionalized selenourea frameworks with potential relevance in coordination chemistry, medicinal chemistry and materials science [176].

The predominant method for selenourea synthesis involves the reaction of alkyl or aryl isoselenocyanates with primary or secondary amines. In this process, the isoselenocyanate functions as a highly electrophilic intermediate that is rapidly attacked by the nucleophilic amine to yield the corresponding selenourea **32** (61%; Scheme 9, *Eq.3*) [175]. These intermediates are typically not isolated or directly observed, and their presence is inferred from the product formation. Nevertheless, isoselenocyanates have proven to be versatile and reliable precursors, facilitating the preparation of a broad spectrum of selenoureas with diverse structural and electronic features. Their operational simplicity and generality have established this approach as the benchmark method for selenourea synthesis in both research and industrial contexts [175].

[Et_4_N]_2_WSe_4_ is a versatile selenium-transfer reagent widely used in organoselenium chemistry. It was first synthesized by O’Neal S.C. and Kolis J.W. via the reaction of K_2_Se_3_ with W(CO)_6_ and Et_4_NBr [177]. Since then, Chandrasekaran and co-workers have extensively applied this reagent in the synthesis of selenoureas [178]. In particular, their group developed a one-pot protocol for the preparation of *N,N*-dimethylselenoureas **33** (50–77%) under mild conditions by reacting primary or secondary amines with Viehe’s iminium salt (phosgene iminium chloride) in the presence of [Et_4_N]_2_WSe_4_ (Scheme 9, *Eq.4*) [179]. The proposed mechanism involves nucleophilic addition of the amine to Viehe’s iminium salt, forming a reactive intermediate in which one chloride is displaced by the amine. Subsequently, [Et_4_N]_2_WSe_4_ transfers selenium to the intermediate, generating the corresponding selenourea with concomitant elimination of WSe_3_. This method provides a mild, efficient, and general approach to selenourea synthesis with a broad substrate scope and operational simplicity [180].

Cycloselenoureas and their derivatives can be efficiently synthesized under analogous reaction conditions by employing amines that contain adjacent functional groups, such as amino, hydroxyl, or thiol moieties. Additionally, dithiols and diols can also serve as suitable substrates in the presence of [Me_4_N][SeCF_4_], facilitating the formation of cyclic selenoureas **34** (30–94%; Scheme 9, *Eq.5*) [180]. [Me_4_N][SeCF_4_] serves as a stable, convenient, and reactive selenium-transfer reagent. This approach enables the construction of a variety of structurally diverse cyclic selenoureas, highlighting the reagent’s versatility and the method’s tolerance toward multiple nucleophilic functionalities.

#### 2.8.2. Green Synthesis of Selenoureas

**Scheme 10:**
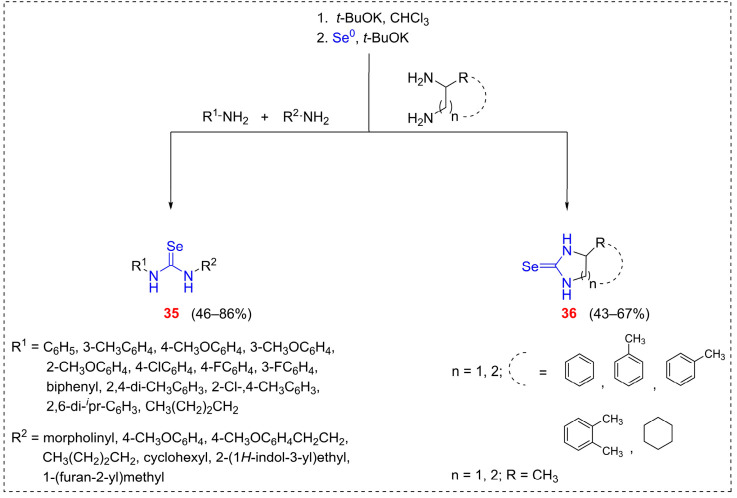
Green synthesis of selenoureas.

Li et al. reported an efficient one-pot, four-component method for the synthesis of selenoureas **35** (46–86%; Scheme 10) using elemental selenium, chloroform, and amines under mild conditions [181]. This protocol allows the straightforward synthesis of unsymmetrical selenoureas by combining selenium powder, chloroform, and two different amines in the presence of a base. The method is operationally simple, practical, and proceeds under mild reaction conditions, avoiding the need for preformed selenium reagents. Using this approach, the authors successfully synthesized thirty-three new unsymmetrical selenoureas **35** and cycloselenoureas **36** (43–67%) by employing appropriate diamines [181].

### 2.9. Selenoketones

Selenoketones (Figure 3, Table 1) are selenium analogs of ketones, characterized by a C=Se functional group. The C=Se bond is significantly more reactive than C=S or C=O, which influences both their chemical behavior and reactivity patterns in synthetic transformations. Due to their high reactivity, selenoketones are rarely isolated and often tend to oligomerize, making their handling and storage challenging. When stabilized, selenoketones provide valuable insight into selenium’s electronic effects in π-systems and can serve as versatile intermediates for constructing complex organoselenium frameworks [182].

Selenoketones exhibit structural similarity to both thio- and oxygen-containing ketones [97,163]. The polarizable C=Se bond renders selenoketones highly reactive in nucleophilic addition, cyclization, and selenium-transfer reactions, facilitating the synthesis of diverse organoselenium scaffolds such as selenazoles, selenoesters, and benzisoselenazolones [97,160,163].

Most reported routes require harsh reaction conditions, elevated temperatures, air-sensitive reagents, or stoichiometric amounts of selenium-containing auxiliaries and reducing agents, which conflict with green chemistry principles [20,169]. The use of inert atmospheres, nonrenewable solvents, and metal-based selenating agents further limits the sustainability of these approaches [21,163]. Consequently, there is a clear need for mild, catalytic, and solvent-friendly methods for the preparation of selenoketones that employ safer reagents and operationally simple protocols, thereby minimizing toxic byproducts, utilizing recyclable catalysts, and incorporating safer selenium sources. Addressing this gap represents a critical area for future research, aligning selenoketone chemistry with the principles of sustainable and green organoselenium synthesis [20,169].

#### Conventional Synthesis of Selenoketones

**Scheme 11:**
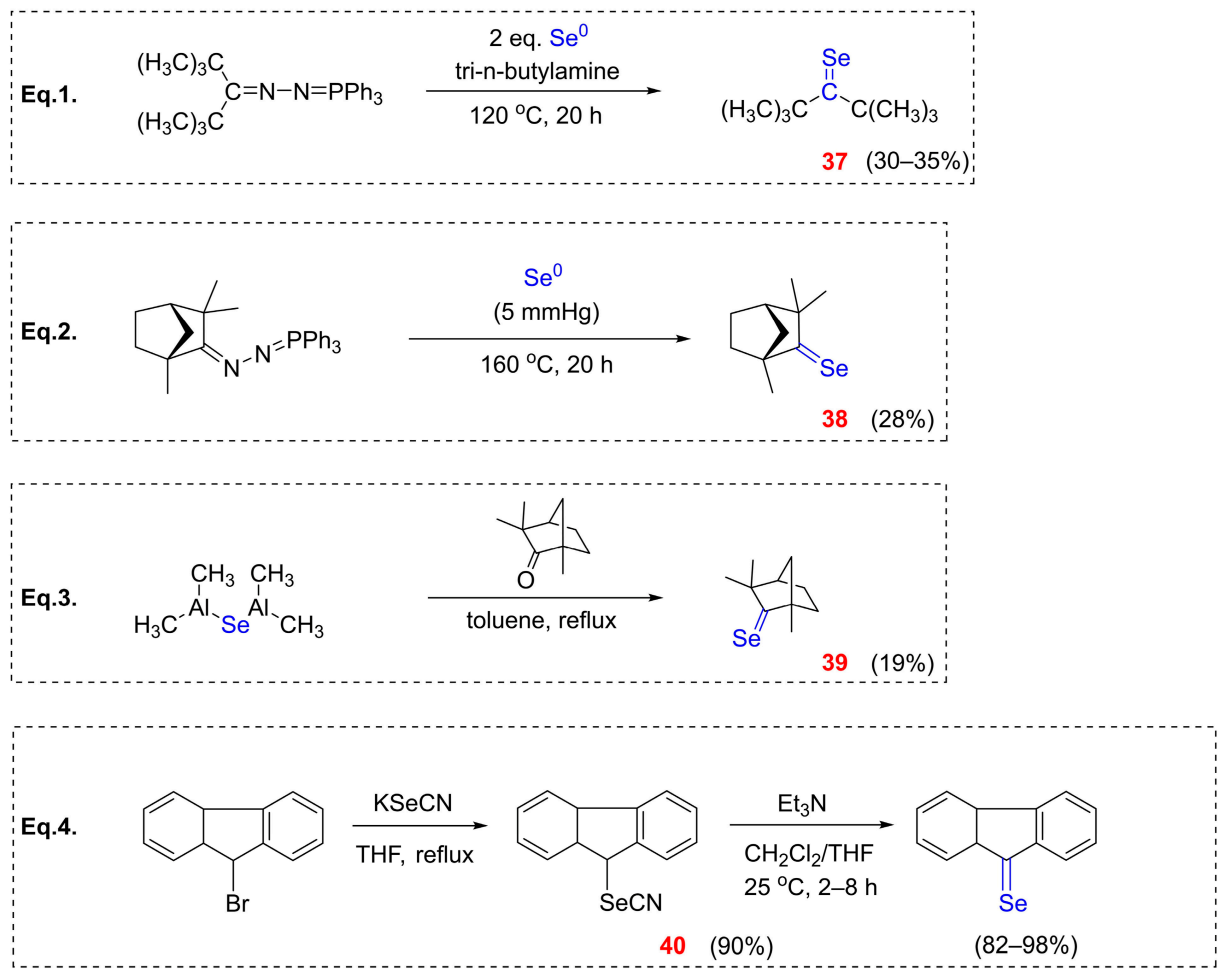
Conventional synthesis of selenoketones.

Thomas Back et al. reported an early and notable synthesis of selenoketones involving the thermal reaction of di-*t*-butyl ketone triphenylphosphoranylidene hydrazone with elemental selenium in the presence of a catalytic amount of tri-*n*-butylamine (Scheme 11, *Eq.1*) [182]. When the reaction mixture was heated under a nitrogen atmosphere at 120 °C, the corresponding blue di-*t*-butyl selenoketone **37** (30–35%; Scheme 11, *Eq.1*) was formed and subsequently isolated by distillation [182]. This work represented one of the first successful preparations of a monomeric selenoketone and provided important insight into the synthesis and properties of these highly reactive selenium-containing carbonyl analogues [182].

Similarly, the (-)-fenchone derivative was heated to 160 °C under reduced pressure with continuous removal of volatile byproducts, yielding (-)-selenofenchone **38** (28%; Scheme 11, *Eq.2*) as blue crystals, which were recrystallized from ether at −30 °C. The reaction proceeds via thermal cleavage of the selenium-containing auxiliary attached to the substrate, allowing the formation of the highly polarizable C=Se bond. The reduced pressure continuously removes volatiles, shifting the equilibrium toward selenoketone formation, while the rigid bicyclic fenchone framework preserves stereochemistry. The blue coloration of the crystals reflects the extended π-conjugation and electronic effects of the selenium atom. This transformation exemplifies the unique reactivity of selenoketones, where sterically and electronically stabilized intermediates can be generated under thermal conditions and isolated through careful recrystallization [182].

In parallel historical developments, the use of bis(dimethylaluminum) selenide emerged as a more general strategy for the conversion of ketones into the corresponding selenoketones **39** (19%; Scheme 11, *Eq.3*) [182]. This reagent was shown to efficiently effect selenium insertion into carbonyl compounds and initially represented a significant advance in selenocarbonyl chemistry. Subsequent studies expanded its application to a broader range of selenocarbonyl derivatives and evaluated the overall utility of bis(dimethylaluminum) selenide as a selenating agent in organic synthesis. Despite its effectiveness, this approach typically afforded moderate yields and required the use of air-sensitive organoaluminum reagents, which limited its practicality and prompted further exploration of alternative and more sustainable synthetic routes to selenoketones [182].

The characterization of most selenoketones has traditionally relied on such trapping experiments, although sterically hindered systems such as selenofenchone have been directly isolated as blue crystalline products from fenchone under reflux, establishing bis(dimethylaluminum) selenide as a powerful reagent for accessing selenoketones and reinforcing Diels–Alder cycloaddition as a valuable probe of their reactive behavior [182].

A major advance in selenocarbonyl chemistry was achieved with the development of a novel route to selenoketones from ketones using bis(dimethylaluminum) selenide, which enables the in situ conversion of carbonyl compounds into otherwise elusive selenoketones [183]. Rather than isolating the transient selenoketones, their formation was elegantly demonstrated through trapping reactions, particularly Diels–Alder cycloadditions with 1,3-dienes [183]. For example, the preparation of selenofluorenone and its subsequent cycloaddition reactivity can be achieved efficiently through a two-step sequence starting from fluorenyl bromide, as reported by Meinke et al. [184]. Fluorenyl bromide undergoes nucleophilic substitution with potassium selenocyanate under reflux in tetrahydrofuran to afford fluorenyl selenocyanate in high yield. Subsequent transformation to selenofluorenone is accomplished by the controlled addition of triethylamine in a dichloromethane/tetrahydrofuran mixture at ambient temperature over 2–8 h, providing the target selenium-containing selenoketone **40** (90%; Scheme 11, *Eq.4*) in good yield.

### 2.10. Selenocyanates

Selenocyanates (Figure 3, Table 1) are formed by nucleophilic substitution, which typically involves the replacement of a leaving group X in an organic compound R-X by a selenolate ion R^1^-Se^⊝^ or selenocyanate ion SeCN^⊝^ and act as analogs of thiocyanates [185]. These compounds are used as intermediates in organic synthesis and exhibit electrophilic reactivity at the selenium center [21]. The unique electrophilic nature of the selenocyanate group allows interaction with thiol groups in enzymes and proteins, influencing redox homeostasis and immune responses [163]. While promising, the biological effects of selenocyanates depend on their chemical structure and dosage, necessitating further research to optimize their safety and efficacy.

#### 2.10.1. Conventional Synthesis of Selenocyanates

**Scheme 12:**
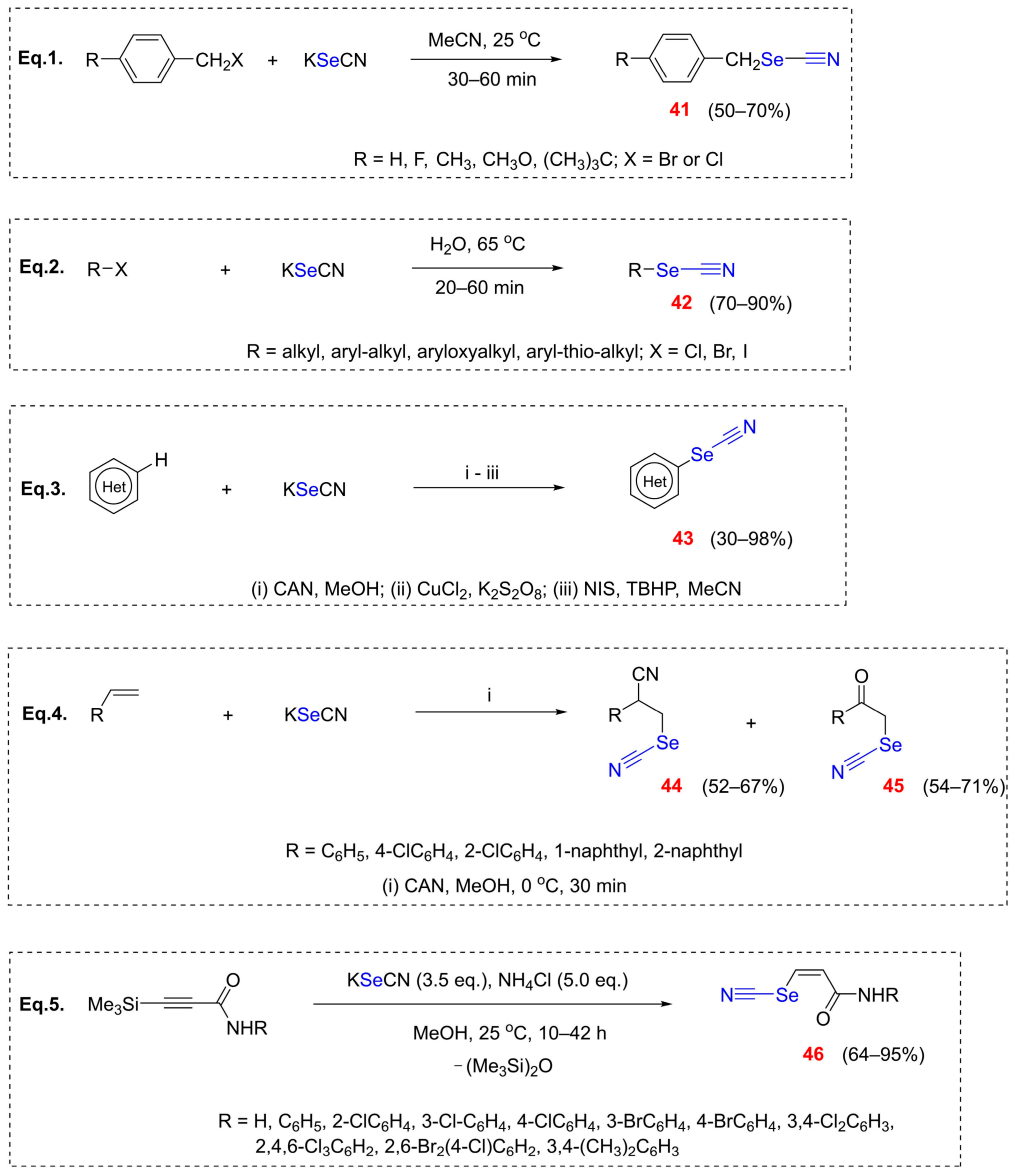
Conventional synthesis of selenocyanates.

In exploring practical methods for introducing selenocyanate functionality, Jacob and co-workers developed a notably efficient and operationally simple procedure for the synthesis of benzyl selenocyanates **41** (50–70%; Scheme 12, *Eq.1*) [186]. The success of this approach hinges on the judicious choice of acetonitrile as reaction medium, which not only dissolves the benzylic halide substrates and potassium selenocyanate effectively but also leaves inorganic by-products such as potassium chloride and potassium bromide as insoluble solids that precipitate out, providing a convenient visual indicator of reaction progress. Critically, the low nucleophilicity of acetonitrile prevents its competitive reaction with the selenocyanate anion, avoiding side-product formation and allowing the substitution to proceed directly at ambient temperature, typically completing in under an hour.

Following simple work-up and a single recrystallization step, analytically pure benzyl selenocyanates **41** (Scheme 12, *Eq.1*) are obtained without the need for chromatographic purification, streamlining the preparation of this versatile class of organoselenium compounds from readily available benzylic bromides [186].

The most common strategies for the preparation of organoselenocyanates **42** (70–90%; Scheme 12, *Eq.2*) involve nucleophilic substitution of potassium selenocyanate with a wide range of electrophilic precursors, including alkyl, aryl, aryl–alkyl, aryloxyalkyl, and aryl–thio–alkyl halides [187]. In this transformation, the halide leaving group (Cl, Br, or I) is displaced by the selenocyanate anion in aqueous media at high temperature, generally requiring 20–60 min for completion. This method provides a straightforward and versatile route to organoselenocyanates, which are valuable intermediates for the synthesis of a variety of selenium-containing compounds, including selenoureas, selenoesters, and heterocyclic selenium scaffolds.

Direct selenocyanation of arenes and heteroarenes is generally achieved through oxidative or catalytic procedures employing reagents such as cerium(IV) ammonium nitrate, copper chloride in combination with potassium persulfate and tetrabutylammonium iodide, or *tert*-butyl hydroperoxide with *N*-iodosuccinimide, which collectively enable the formation of aromatic and heteroaromatic selenocyanates **43** (30–98%; Scheme 12, *Eq.3*) [188]. By contrast, methods that effect the addition of inorganic selenocyanate across carbon–carbon multiple bonds in alkenes or alkynes remain comparatively scarce. Broadening the scope of such additions would significantly enhance the synthetic utility of selenocyanation chemistry by facilitating access to previously underexplored classes of organoselenium frameworks, including vinyl selenocyanates.

Several illustrative examples highlight the limited but evolving scope of selenocyanate addition to unsaturated substrates. Transition-metal-mediated reactions of potassium selenocyanate with substituted styrenes under aerobic conditions, employing catalysts such as cerium(IV) ammonium nitrate or copper(II) salts, including copper(II) chloride and copper(II) bromide, have been shown to yield saturated bis(selenocyanate) **44** (52–67%) and oxoselenocyanate derivatives **45** (54–71%; Scheme 12, *Eq.4*) [187,189]. An efficient and conceptually distinct strategy has been introduced by Hassanpour et al. for the synthesis of a new class of (*Z*)-3-amino-3-oxo-1-propenyl selenocyanates **46** (64–95%; Scheme 12, *Eq.5*) [188]. This transformation relies on the reaction of potassium selenocyanate with 3-trimethylsilyl-2-propynamides in methanol, promoted by ammonium chloride under ambient conditions. The procedure is notable for its high efficiency, excellent stereoselectivity, and consistently good yields. Although terminal propynamides can also participate in the reaction, their limited commercial availability renders 3-trimethylsilyl-substituted analogues that are readily accessible from 3-trimethylsilylpropiolic acid a more practical and versatile choice of substrate for this methodology.

#### 2.10.2. Green Synthesis of Selenocyanates

**Scheme 13:**
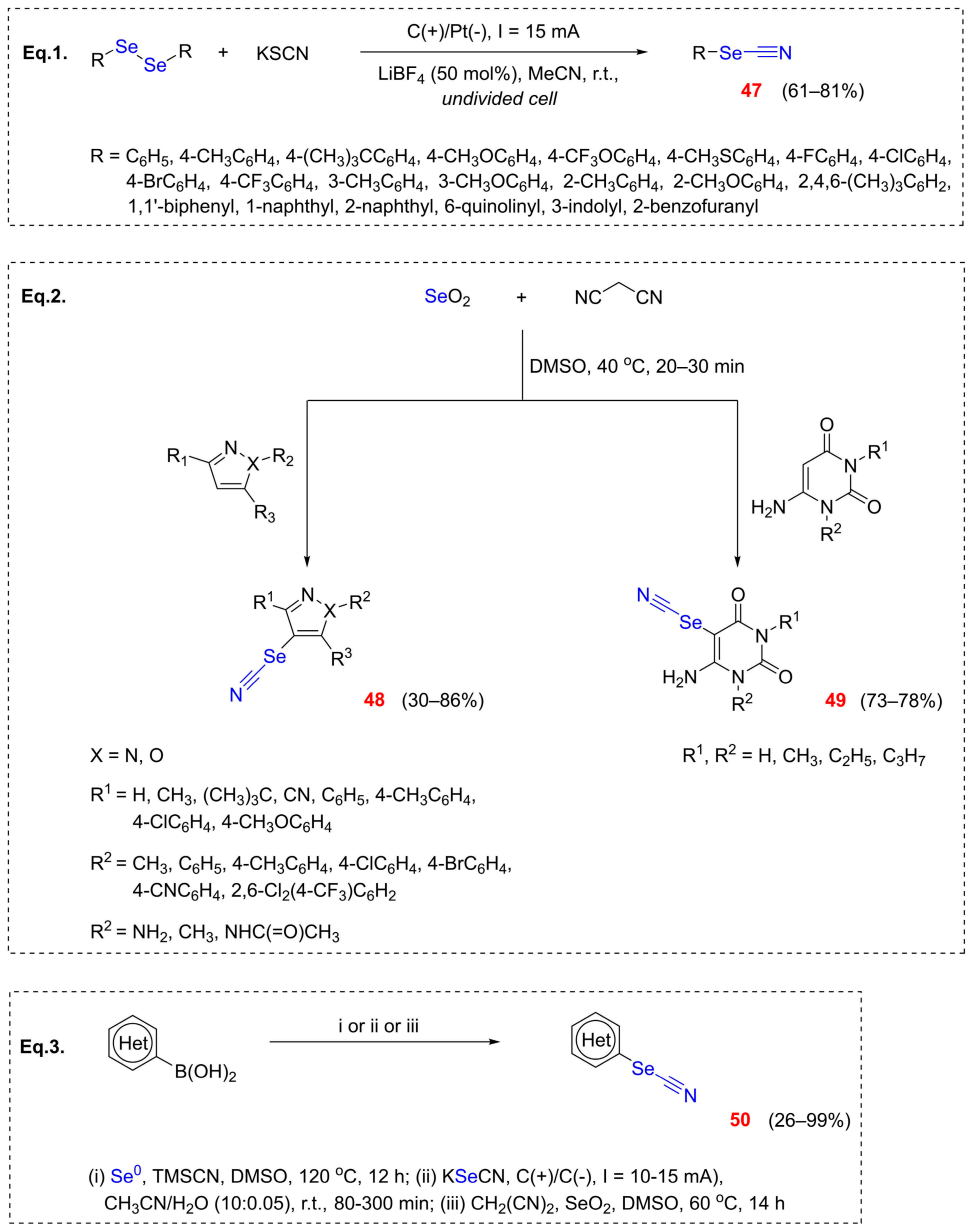
Green synthesis of selenocyanates.

In a recent study, He et al. reported an efficient electrochemical approach to the synthesis of aryl selenocyanates via paired electrolysis of diaryl diselenides in the presence of a thiocyanate source [190]. Under mild, catalyst-free conditions, a broad range of diaryl diselenides bearing electron-donating and electron-withdrawing substituents underwent smooth conversion to the corresponding aryl selenocyanates **47** (61–81%) in generally good to excellent yields (Scheme 13, *Eq.1*) [190]. The reaction exhibited high functional-group tolerance and proceeded efficiently at ambient temperature without the need for external chemical oxidants, highlighting the advantages of electrochemical activation. Notably, the paired electrolysis strategy enables simultaneous anodic and cathodic processes, enhancing energy efficiency while minimizing by-product formation. Overall, the summarized substrate scope demonstrates the robustness and synthetic versatility of this electrochemical protocol for accessing structurally diverse aryl selenocyanates in a sustainable manner. The methodology reported by Ali et al. offers a practical and versatile route to both selenocyanates and selenoethers of amino pyrazoles and amino uracils through the in situ generation of triselenium dicyanide from readily available malononitrile and selenium dioxide (Scheme 13, *Eq.2*). Systematic solvent screening identified dimethyl sulfoxide as uniquely effective in promoting this transformation, likely due to its ability to stabilize reactive selenium species and facilitate cyanide transfer.

Optimization of the reaction conditions established that combining equimolar 5-amino-3-methyl-1-phenyl-1*H*-pyrazole with 1.5 equivalents of malononitrile and 3 equivalents of selenium dioxide in dimethyl sulfoxide at 40 °C delivers the selenocyanated product derivatives **48** (30–86%) and **49** (73–78%; Scheme 13, *Eq.2*) in their optimal yields [191]. This procedure thus exemplifies a straightforward, mild, and operationally simple approach to functionalized organoselenium compounds via a transient triselenium dicyanide intermediate.

Recent advances in the deborylative functionalization of aryl boronic acids have provided versatile and efficient routes for the synthesis of selenocyanate groups. Early investigations (route i) demonstrated the feasibility of converting aryl boronic acids into selenocyanates 50 (26–99%; Scheme 13, *Eq.3*) via deborylative cyanation under oxidative conditions [192]. These studies established the foundational understanding that the boronate group can be selectively transformed into a reactive aryl radical or cation intermediate. Building on this foundation, more recent work (Scheme 13, *Eq.3*, route ii) has expanded the substrate scope and optimized reaction conditions, allowing for efficient formation of diverse aryl and heteroaryl selenocyanates 50 with improved yields and regioselectivity [193]. Notably, an electrochemical strategy (Scheme 13, *Eq.3*, route iii) now enables deborylative seleno- and thiocyanation 50 under catalyst- and oxidant-free conditions [192]. In this approach, anodic oxidation of the arylboronic acid generates an aryl radical intermediate that subsequently reacts with KSeCN at the cathode, producing the corresponding aryl selenocyanate. This electrochemical protocol proceeds under mild conditions, exhibits broad functional-group tolerance, and avoids the use of stoichiometric oxidants or transition-metal catalysts, aligning with principles of green and sustainable chemistry.

Collectively, these studies illustrate the growing synthetic utility of deborylative transformations, highlighting how the combination of traditional and electrochemical methods provides a modular and efficient approach to access organoselenium compounds from readily available boronic acid precursors.

### 2.11. Selenoamides

Selenoamides represent an important subclass of organoselenium compounds in which selenium is incorporated into an amide framework, and their synthesis has attracted increasing attention in organic chemistry due to the unique reactivity of the C=Se bond. Several efficient synthetic strategies have been developed to access selenoamides, including the conversion of amides and lactams into their corresponding selenoamides using selenium transfer reagents such as tetraethylammonium tetraselenotungstate, which enable direct carbonyl chalcogen exchange under mild conditions [178]. Alternative approaches to selenoamide synthesis involve selenium dioxide-mediated oxidative amidation of carbonyl precursors with amines: for example, selenium dioxide promotes the oxidative coupling of arylglyoxals with secondary amines to form α-ketoamides in a one-pot process, demonstrating the utility of selenium dioxide as an oxidant in constructing carbon–nitrogen bonds under mild conditions [194]. In addition, ring-opening reactions of heterocyclic precursors such as 2-oxazolines with selenium nucleophiles have been reported as a reliable route to β-selenoamides with good regio- and stereocontrol [178]. These methodologies collectively highlight the versatility of selenium reagents in amide functionalization and establish selenoamides as valuable intermediates in organoselenium synthesis [195].

#### 2.11.1. Conventional Synthesis of Selenoamides

**Scheme 14:**
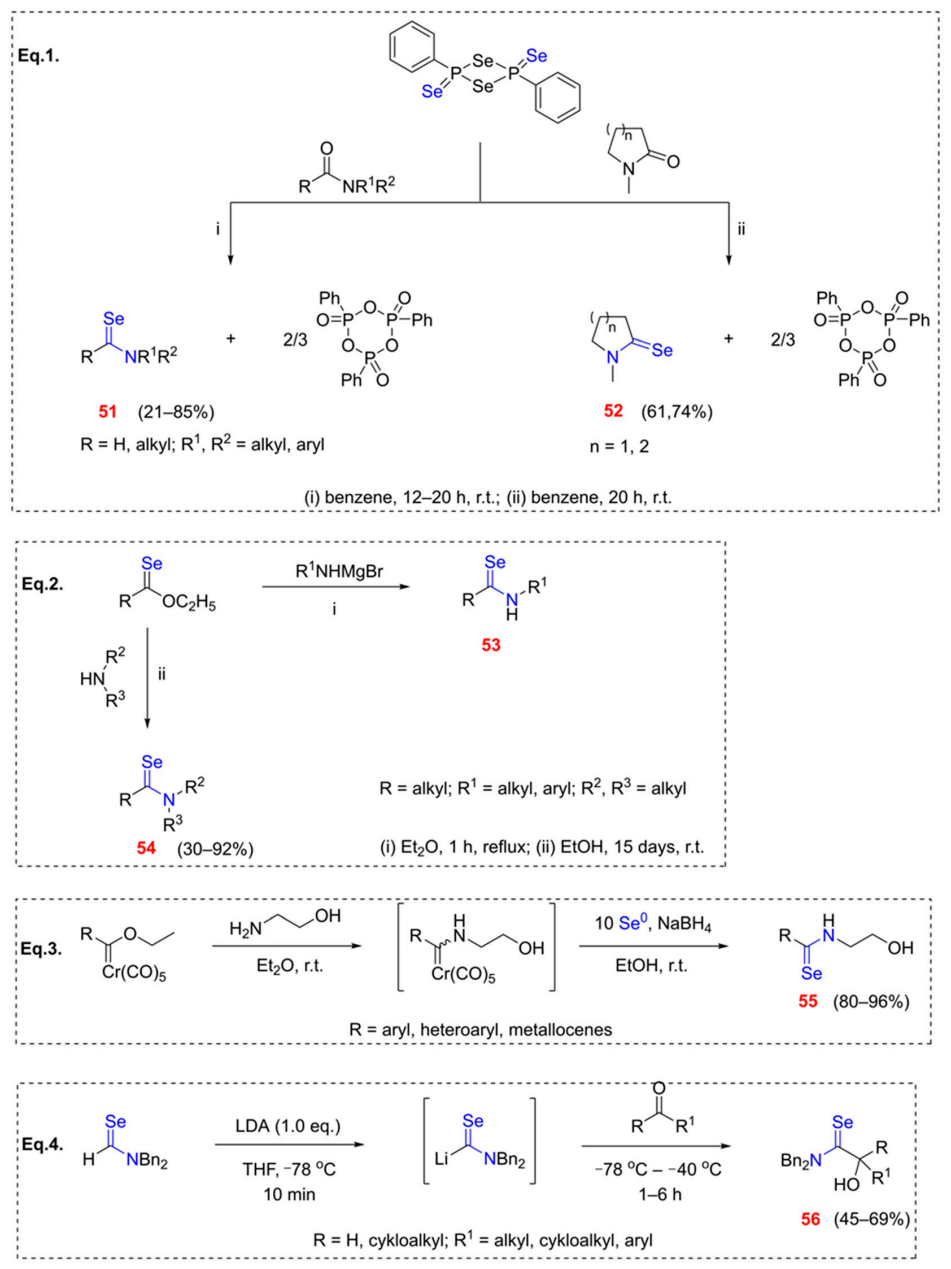
Conventional synthesis of selenoamides.

As demonstrated by Bethke et al., the selenium analogue of Lawesson’s reagent, 1,3-di-selena-2,4-diphosphetane-2,4-diselenide, serves as an efficient selenation reagent for converting amides into *N*,*N*-disubstituted selenoamides via O/Se exchange [196]. Lawesson’s reagent’s role as an efficient selenium transfer reagent via an O/Se exchange process was first demonstrated by Bhattacharyya and Woollins [197]. Scheme 14, *Eq.1*, presents the direct synthesis of *N*,*N*-disubstituted selenoamides [196]. In this transformation, the reaction allows the selective replacement of the amide carbonyl oxygen with a selenium moiety under mild and practical conditions, reflecting a selenation process analogous to the well-known thionation achieved with Lawesson’s reagent. The selenium–Lawesson reagent acts as a selenium transfer agent, activating the amide carbonyl and promoting the formation of a strong P=O bond as the thermodynamic driving force for the exchange. As a result, the C=O group is converted into a C=Se functionality without affecting the *N*-substituents, enabling efficient access to a range of *N*,*N*-disubstituted selenoamides **51** (21–85%) and **52** (61%, 74%) in moderate to good yields (Scheme 14, *Eq.1*) [196].

A versatile method for synthesizing aliphatic and aromatic selenoesters and transforming them into selenoamides has expanded access to this underexplored class of compounds. Early approaches to selenoamide synthesis involved harsh or limited methods, such as the reaction of phosphorus pentaselenide with tertiary amines (e.g., *N*,*N*-dimethylbenzylamine) or the transformation of 5-unsubstituted 1,2,3-selenadiazoles with amines, as reported by Yalpani and Malek-Yazdi [198]. Building on these foundations, the availability of selenoesters enabled a more direct and systematic entry into selenoamides **53** and **54** (30–92%), as presented in Scheme 14, *Eq.2* [199]. Cohen suggested that the ready accessibility of selenoesters offers a convenient and general entry to *N*-mono- and *N,N*-disubstituted selenoamides, providing a practical foundation for further development of selenoamide synthesis. In this context, aliphatic selenoesters were shown to react with primary alkylamide magnesium bromides in diethyl ether to afford *N*-monosubstituted selenoamides **53** (Scheme 14, *Eq.2*, route i), whereas prolonged reaction with secondary amines led to *N*,*N*-disubstituted selenoamides **54** (Scheme 14, *Eq.2*, route ii). However, reactions with primary amines often produced competing imido esters and hydrogen selenide byproducts, highlighting the need for milder and more applicable methods for selenoamide synthesis.

As reported by García-López et al., the synthesis outlined in their work involves the preparation of ethoxycarbene and aminocarbene Fischer carbene complexes, which serve as key precursors for accessing selenocarbonyl compounds [200]. Following aminolysis to generate the aminocarbene complexes, selenative demetalation was carried out using a Se/NaBH_4_ reagent system previously developed by the authors, enabling efficient conversion to the corresponding arylselenoamide derivative **55** (80–96%; Scheme 14, *Eq.3*) [200]. The reactions proceeded rapidly, typically within 15–45 min, affording the products as yellow to red solids in moderate to good yields depending on the carbene substituents. To further improve efficiency, the authors implemented a two-step one-pot aminolysis–demetalation procedure, which increased overall yields to as high as 80% from readily available aromatic starting materials. Notably, no selenol byproducts were observed, and the preservation of hydroxyl groups in all products demonstrates the high chemoselectivity and functional-group tolerance of this methodology, highlighting Fischer carbene complexes as versatile intermediates for the mild synthesis of selenoamides [200].

Murai et al. reported a method for the synthesis of α-hydroxy and α-oxo selenoamides via nucleophilic selenocarbamoylation of carbonyl compounds, highlighting the replacement of the carbonyl oxygen with selenium to generate selenocarbonyl compounds with distinct electronic properties and visible-light absorption, though generally lower stability than their oxygen analogues [201]. The authors noted that the stability of these species is strongly influenced by substitution at the C=Se moiety, with bulky groups or adjacent heteroatoms (O, N, S) enhancing isolability. In this approach, selenocarbamoyllithiums are generated by deprotonation of selenoformamides with lithium diisopropylamide, serving as nucleophilic “umpolung” reagents capable of adding to ketones and aldehydes to furnish α-hydroxy and α-oxo selenoamides, including the first isolated α-hydroxy selenoamide. Product yields were highly dependent on reaction temperature, stoichiometry, and substrate sterics, with sterically demanding ketones and aromatic aldehydes generally affording higher yields. This methodology underscores the utility of selenocarbamoyllithiums as versatile intermediates for accessing structurally diverse and functionally rich selenoamides **56** (45–69%; Scheme 14, *Eq.4*) [201].

#### 2.11.2. Green Synthesis of Selenoamides

**Scheme 15:**
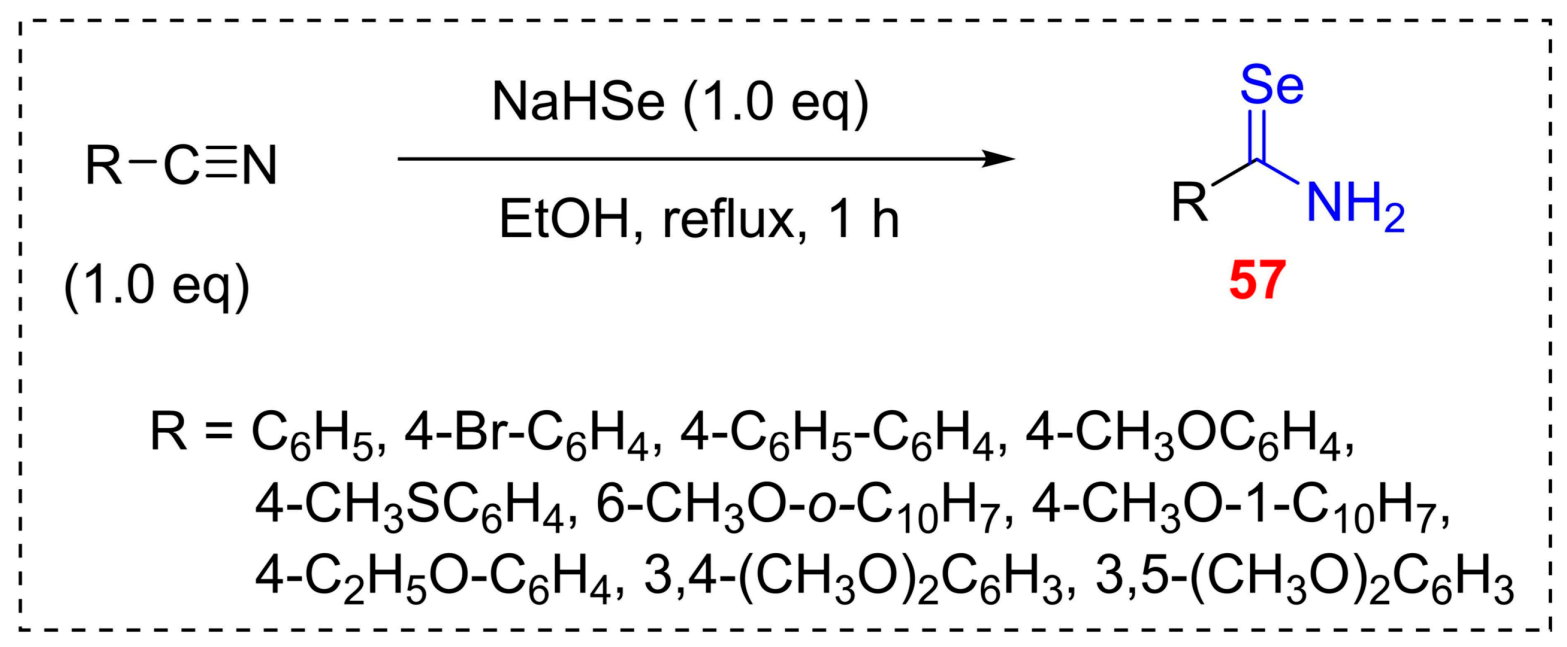
Green Synthesis of Selenoamides.

Scheme 15 highlights the synthesis of primary selenoamides **57** via the direct reaction of aromatic nitriles with sodium hydroselenide in ethanol under reflux conditions for 1 h [202,203]. The reaction proceeds smoothly to provide the corresponding selenoamides in high yield, highlighting the efficiency of this transformation across a broad range of aromatic and substituted aromatic nitriles, including halogenated, alkylated, and methoxylated derivatives. The selenoamide serves as a highly versatile intermediate, readily participating in cyclization and other transformations to access diverse selenium-containing heterocycles. The broad substrate compatibility further emphasizes the role of selenoamides as versatile and valuable building blocks in organoselenium chemistry [202,203].

### 2.12. Seleniranes

Seleniranes are three-membered heterocycles consisting of one selenium atom and two carbon atoms, making them the selenium analogues of oxiranes and thiiranes (Figure 3, Table 1) [106,118]. Their high ring strain and the large, polarizable selenium atom render them highly reactive and generally unstable, prone to ring-opening reactions and facile decomposition under thermal or nucleophilic conditions [204,205,206]. As a result, their chemistry has been explored primarily in mechanistic studies within organoselenium chemistry rather than in practical synthetic applications [118,207].

Seleniranes can be synthesized via the addition of selenium or selenating species across carbon–carbon multiple bonds or through intramolecular cyclization reactions. Due to their high reactivity, isolation typically requires sterically bulky substituents or low-temperature conditions [118,208]. Direct studies on the health benefits of seleniranes are limited; however, their chemical reactivity indicates potential as anticancer and antimicrobial agents by inducing oxidative stress or modifying critical biomolecules in pathogens or cancer cells [163]. Furthermore, seleniranes are valuable synthetic intermediates for preparing more complex selenium-containing bioactive molecules, offering a platform for drug discovery targeting oxidative stress-related diseases [106]. Thus, further research is needed to fully elucidate their biological roles and therapeutic potential.

#### Conventional Synthesis of Seleniranes

**Scheme 16:**
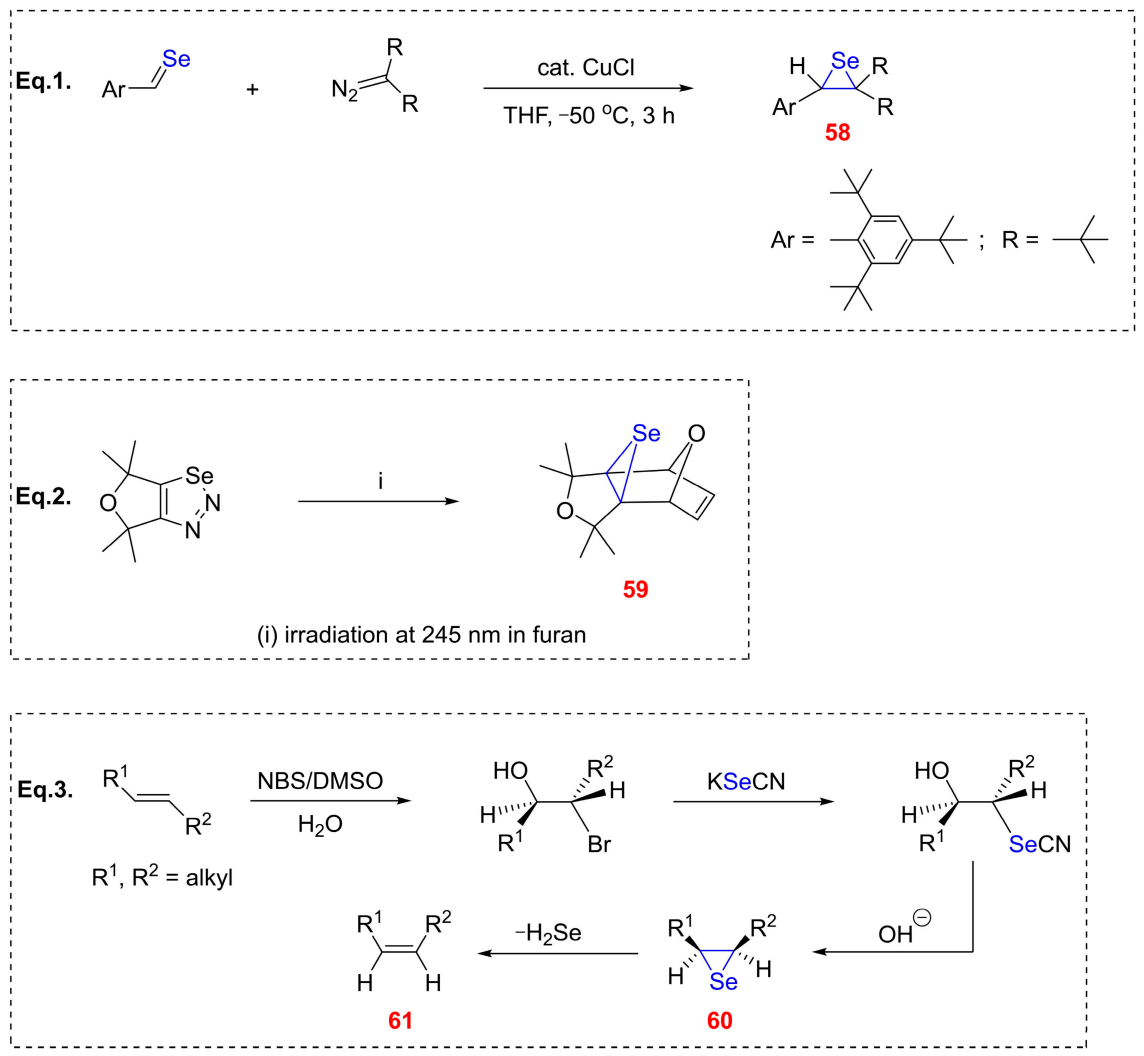
Conventional synthesis of seleniranes.

In the study by Watanabe et al., the highly hindered selenoaldehyde 2,4,6-tri-*tert*-butylselenobenzaldehyde was reacted with sterically congested diazo compounds, including di-*tert*-butyldiazomethane and 2-diazo-1,1,3,3-tetramethylindan, under low-temperature conditions in the presence of catalytic copper(I) chloride [209]. This reaction proceeds via nucleophilic addition of the diazo species to the electrophilic carbon–selenium bond of the selenoaldehyde, followed by ring closure to form three-membered selenium-containing heterocycles, seleniranes **58** (Scheme 16, *Eq.1*) [209]. The significant steric bulk on both the aldehyde and diazo substrates is critical for stabilizing these strained intermediates, allowing their isolation and characterization. The resulting seleniranes exhibit enhanced stability relative to less hindered analogues and serve as valuable models for studying the electronic and steric effects of selenium in small-ring systems, highlighting the potential of sterically protected selenoaldehydes as versatile precursors for constructing novel selenium-containing heterocycles.

The photolysis of a 1,2,3-selenadiazole under UV irradiation (*hv* = 245 nm) in furan generates a highly strained selenirane **59** (Scheme 16, *Eq.2*) [210]. The selenadiazole undergoes photochemical nitrogen extrusion to produce the three-membered Se–C–C ring. Subsequent treatment with tri-methylphosphine [(Me_3_N)_3_P] in chloroform stabilizes the selenirane, likely via coordination or trapping, yielding a stable selenirane derivative [207]. This transformation illustrates a photochemically driven approach to three-membered selenium heterocycles, highlighting the unique reactivity of selenadiazoles as precursors for isolable seleniranes under mild conditions. The bulky substituents in this system help stabilize the otherwise highly strained selenirane, enabling its characterization and subsequent utilization in organoselenium synthesis.

In the third step of the formation of selenirane **60** (Scheme 16, *Eq.3*), the halohydrin intermediate containing a bromine atom reacts with potassium selenocyanate to undergo nucleophilic substitution, whereby the SeCN^⊝^ anion displaces the bromide to yield a β-hydroxy selenide [211]. Subsequent treatment with a base, typically a hydroxide ion, induces intramolecular cyclization via nucleophilic attack of the hydroxyl group on the selenium atom, resulting in the formation of a three-membered selenirane ring. Finally, elimination of H_2_Se from the intermediate restores the double bond, generating the substituted alkene product. This sequence highlights the utility of seleniranes as versatile intermediates in the synthesis of functionalized alkenes **61** (Scheme 16, *Eq.3*) [211]. It also demonstrates the characteristic nucleophilic displacement, cyclization, and elimination mechanisms typical of selenium-containing three-membered heterocycles **60** (Scheme 16, *Eq.3*) [211]. This methodology also highlights the unique reactivity of selenium, which differs from that of its lighter congeners, sulfur and oxygen, allowing for transformations that are often difficult to achieve with other heteroatoms.

## 3. Comparison of Key Process Parameters for Conventional and Green Synthesis

Green synthetic methods for organoselenium compounds consistently deliver yields that are comparable to, and in many cases exceed, those obtained using conventional protocols, while simultaneously offering substantial environmental and operational benefits. As summarized in Table 2, these greener approaches demonstrate strong performance across a wide range of selenium compound classes, underscoring their efficiency, versatility, and practical viability as sustainable alternatives to traditional methodologies. In the case of divinyl selenides, the use of PEG-400 as a green reaction medium in combination with sodium borohydride enables yields of 74–93%, which are comparable to or higher than those obtained through classical sodium hydroselenide/alkyne reactions performed in dimethyl sulfoxide or aqueous systems. Importantly, the PEG-based protocol operates under milder conditions with shorter reaction times, while avoiding the use of malodorous and hazardous sodium hydroselenide, thereby improving both safety and sustainability [212]. The conventional synthesis of the target compound using acetylene with potassium hydroxide/tin(II) chloride in H_2_O at 105–115 °C for 15 h affords an 80% yield. While the yield is reasonably high, the method requires long reaction times, elevated temperatures, and the use of tin salts, which are toxic and environmentally problematic [213].

Similarly, the synthesis of diarylselenides using copper oxide or Zn in recyclable ionic liquids such as [bmim][PF_6_] affords high yields of up to 96%, clearly surpassing conventional arenediazonium salt methodologies that rely on volatile organic solvents, inert atmospheres, and less environmentally benign selenium sources [157]. In addition to improved yields, ionic liquids offer recyclability and reduced solvent waste, further enhancing the green credentials of these approaches. However, the conventional method employs arenediazonium salts and arylselenols in tetrahydrofuran under nitrogen at room temperature with hypophosphorous acid, providing moderate to good yields of 50–80%. While the reaction conditions are mild, the use of tetrahydrofuran, an organic solvent with environmental and safety concerns, makes this approach less sustainable. Compared with greener alternatives that use H_2_O or solvent-free conditions, this procedure still relies on conventional organic solvents and requires careful handling, highlighting its conventional route rather than fully green characteristics [214].

For alkynyl selenides, aerobic oxidative coupling reactions conducted in PEG-200 at 100 °C achieve yields as high as 90% [215]. Under the same reaction sequence, with 10 min for the SN2 step, one hour with tripotassium phosphate, and two hours with potassium *tert*-butoxide, conventional protocols in dimethylformamide provide significantly lower yields, typically 21–61% [215]. The use of PEG not only enhances reaction efficiency but also allows molecular oxygen from air to act as a green oxidant, which eliminates the need for stoichiometric chemical oxidants and improves the overall sustainability of the process.

Mechanochemical ball-milling strategies for diselenide formation exemplify the advantages of solvent-free green synthesis. These approaches deliver excellent yields of up to 98% while eliminating the need for solvents, high temperatures, and inert gas protection [132]. Such methodologies significantly reduce energy consumption and waste generation, making them particularly attractive for scalable and sustainable synthesis. The conventional synthesis of diselenides typically involves the reduction of elemental selenium with NH_2_NH_2_·H_2_O in the presence of sodium hydroxide in dimethylformamide. The resulting selenide intermediate is then alkylated with benzyl bromide or other alkyl halides to afford the corresponding diselenides in moderate to good yields of 57–88% within two hours. While effective, this method relies on toxic reducing agents and organic solvents, highlighting its conventional nature compared with greener, more sustainable alternatives [216].

Selenoureas can be synthesized under green conditions using potassium carbonate and elemental selenium in a mixed ethanol/tetrahydrofuran solvent at 60 °C, achieving high yields of 70–94% in just two minutes [217]. In contrast, conventional methods using potassium carbonate in acetonitrile require longer reaction times of 10–20 min and give yields ranging from 30 to 94% [161]. The green approach not only reduces reaction time but also employs a more benign solvent system, highlighting the advantages of sustainable synthesis strategies over traditional methodologies. Selenocyanates can be prepared using potassium selenocyanate in H_2_O, giving moderate to good yields of 40–90% [218]. Conventional methods using potassium selenocyanate in organic solvents such as dimethyl sulfoxide or acetonitrile typically afford yields of around 80% [219]. The aqueous approach offers a greener alternative by avoiding organic solvents while still maintaining comparable efficiency.

Comparable benefits are observed for selenoxides and selenones, where green methodologies employing recyclable solvents, aqueous oxidants such as oxone, or solvent-free conditions consistently afford high yields (selenoxides 85–90% and selenones 84–99%) under milder reaction parameters [17,220]. These methods reduce reliance on hazardous reagents and harsh conditions commonly associated with traditional selenium oxidation and functionalization reactions. Selenoxides can be synthesized using potassium superoxide in acetonitrile at 15 °C, providing yields ranging from 45 to 98% [146]. Selenones are typically prepared by oxidizing the corresponding selenides with magnesium monoperoxyphthalate (MMPP) in an ethanol/tetrahydrofuran mixture at 25 °C for 1 to 3 h, affording yields of 63–94% [221]. These methods are considered conventional, as they rely on organic solvents and chemical oxidants at controlled temperatures to achieve the desired products efficiently.

Makhaeva et al. reported the formation of the selenoamide intermediate; however, the study did not provide the isolated yield or detailed characterization of the product, leaving the efficiency and scope of the method somewhat unclear [203]. Nevertheless, their work demonstrated the feasibility of using sodium hydroselenide as a convenient selenium source for amide functionalization, suggesting potential for further optimization and broader application in organoselenium chemistry [203]. The conventional synthesis of selenoamides is typically carried out using a mixture of ethanol (80–95%), Cr(CO)_6_, a primary amine (H_2_N–R), selenium, and sodium borohydride at 25 °C. The reaction proceeds within 1–3 h, leading to the formation of the desired selenoamides under mild conditions.

For selenium nanoparticles (SeNPs), green biosynthetic approaches using plant extracts or microbial systems clearly outperform conventional chemical reduction methods in both yield and sustainability. As shown in Table 2, plant- and microbe-mediated biosynthesis affords SeNPs in high yields (80–98%), exceeding the 50–70% typically achieved via chemical reduction with sodium borohydride [222,223]. Beyond yield enhancement, biosynthetic routes offer additional advantages that are particularly relevant for selenomaterials, including milder reaction conditions, aqueous media, and the elimination of hazardous reducing agents [224]. Moreover, biological systems often act as both reducing and stabilizing agents, producing surface-functionalized nanoparticles with improved stability, controlled size distribution, and enhanced biocompatibility. These features are critical for applications in biomedicine, catalysis, and environmental remediation, underscoring the superiority of green biosynthetic strategies for the sustainable production of selenium nanomaterials [222,223].

## 4. Conclusions

This review provides a comprehensive overview of conventional and green synthetic routes for organoselenium compounds, including classes of selenols, selenides, ebselen, diselenides, selenoureas, selenocyanates, selenoxides, selenones, selenoamides, seleninic acids, selenoketones, seleniranes, thioselenides, and selenium species. The adoption of green chemistry has enabled the development of more sustainable, efficient, and safer synthetic strategies, including solvent-free reactions, aqueous and bio-based solvents, mechanochemical and microwave-assisted techniques, and electrochemical methods.

Among these approaches, electrochemical C–Se bond formation, solvent-free mechanochemical synthesis, and aqueous catalytic protocols appear particularly promising because they can reduce solvent consumption, energy demand, and dependence on hazardous reagents. However, their sustainability should be confirmed through quantitative green chemistry metrics. Green oxidation using benign oxidants in aqueous media and biological methods for producing selenium nanoparticles also offer valuable opportunities. Future integration of these strategies with continuous-flow processing, recyclable heterogeneous catalysts, and in-line reaction monitoring could improve process control, safety, catalyst recovery, and scalability. Although certain green methods, particularly aqueous selenocyanate syntheses, did not consistently achieve higher yields than conventional protocols, they nevertheless provided meaningful environmental and safety advantages.

The advantages of green syntheses over conventional syntheses were illustrated using several representative examples. Diarylselenides synthesized in ionic liquids using CuO or Zn catalysts achieved excellent yields (75–96%) compared with conventional methods (50–80%). PEG-mediated synthesis also improved the preparation of divinyl and alkynyl selenides, with alkynyl selenide yields reaching 90%, substantially exceeding those obtained using dimethylformamide (21–61%). Green oxidation protocols employing oxone in aqueous media provided high yields of selenoxides (85–92%) and selenones (84–99%) under mild room-temperature conditions, demonstrating both efficiency and operational simplicity. Mechanochemical ball-milling methods for diselenide synthesis further highlighted the advantages of sustainable chemistry by reducing reaction times to minutes while maintaining high yields (55–98%). In addition, biosynthetic approaches for SeNPs afforded excellent yields (80–98%) and avoided the harsh reducing agents commonly used in traditional chemical synthesis. On the other hand, several important challenges remain. Many reported methods have been demonstrated only at the laboratory scale, and their broader application will require scalable catalytic systems with low catalyst loadings, high selectivity, broad substrate scope, and reliable recyclability. Safer procedures are also needed for handling selenium reagents and intermediates, particularly volatile, malodorous, toxic, or air-sensitive species. Continuous-flow and closed-system technologies may help minimize operator exposure and improve the containment and treatment of selenium-containing waste. Moreover, high yield alone does not establish the sustainability of a process. Future studies should apply standardized metrics such as atom economy, process mass intensity, E-factor, Eco-Scale, Green Analytical Procedure Index, and life cycle assessment to enable meaningful comparisons between conventional and green protocols [225,226,227].

Further exploration of biological activity is equally important. Future work should therefore connect synthetic innovation with systematic biological evaluation and pharmacokinetic and toxicological studies. Addressing these challenges will be essential for translating green organoselenium chemistry from laboratory-scale demonstrations into safe, scalable, and practically useful pharmaceutical, biomedical, and industrial applications.

## Figures and Tables

**Table 1:** Categories of organoselenium compounds.

Compound Name	General Formula	Class of Compounds	References
Selenols	R–SeH	Thiol/alcohol analogs	[32,96]
Primary selenols	R–CH_2_–SeH	Analogs of primary alcohols/thiols	[32,96]
Secondary selenols	R_2_–CH–SeH	Analogs of Secondary alcohols/thiols	[32,96]
Tertiary selenols	R_3_–C–SeH	Analogs of tertiary alcohol frameworks	[32,96]
Ebselen	C_13_H_9_NOSe	Heterocyclic selenides	[57,97,98]
Selenides	R–Se–R^1^	Ether analogs	[99]
Diselenides	R–Se–Se–R^1^	Disulfide analogs	[99]
Selenoxides	R–Se(=O)–R^1^	Sulfoxide analogs	[100]
Selenones	R–SeO_2_–R^1^	Sulfone analogs	[101]
Seleninic acids	R–SeO_2_H	Sulfinic acid analogs	[96]
Selenomethionine	C_5_H_11_NO_2_Se	Amino acid analogs	[102,103]
Selenocysteine	C_3_H_7_NO_2_Se	Amino acid analogs	[97,103]
Selenourea	R–NH–C(=Se)–NH–R^1^	Urea analogs	[103]
Selenoketones	R_2_–C=Se	Ketone analogs	[99]
Selenocyanates	R–SeCN	Thiocyanate analogs	[104]
Selenoamides	R-C(=Se)-NR^1^R^2^	Amide analogs	[105]
Seleniranes	C_2_H_4_Se	Three-membered cyclic selenides	[106]
Thioselenides	R–Se–S–R^1^	Mixed chalcogen compounds	[99]

Note: “R” in these structures denotes an organic group, typically an alkyl, aryl, or other organic substituent.

**Table 2:** Comparison of green and conventional synthesis yields for various organoselenium compounds.

Compound Type	Green Synthesis Yield (%)/Conditions	Conventional Synthesis Yield (%)/Conditions	References
Divinyl selenides	74–93% (PEG-400, NaBH_4_, 60 °C, 5 h)	80% (acetylene + KOH/SnCl_2_/H_2_O, 105–115 °C, 15 h)	[212,213]
Diarylselenides	75–96% (CuO or Zn in [bmim][PF_6_])	50–80% (H_3_PO_2_, arenediazonium salts with arylselenols, THF, N_2_, 25 °C)	[157,214]
Alkynyl selenides	90% (PEG 200, O_2_, 100 °C, 10 min (S_N_2), 1 h K_3_PO_4_, 2 h t-BuOK)	21–61% (DMF, O_2_, 100 °C, 10 min (S_N_2), 1 h K_3_PO_4_, 1 h t-BuOK)	[215]
Diselenides	55–98% (aryl and alkyl halides, Mg (1.0 equiv), LiCl (1.5 equiv), THF, ball-milling, 30 Hz, 15 min)	57–88% (Se, NH_2_NH_2_·H_2_O, NaOH, DMF, 2 h, BnBr)	[132,216]
Selenoureas	70–94% (K_2_CO_3_/Se, EtOH/THF, 60 °C, 2 min)	30–94% (K_2_CO_3_, CH_3_CN, 10–20 min)	[180,217]
Selenocyanates	40–90% (KSeCN, H_2_O)	80% (KSeCN in DMSO or CH_3_CN)	[218,219]
Selenoxides	85–92% (oxone_,_ THF/H_2_O_2_, 25 °C, 2 h)	45–98% (KO_2_, MeCN, −15 °C)	[165,220]
Selenones	84–99% (oxone, H_2_O_,_ 25 °C, 2 h)	63–94% (EtOH/THF, MMPP, 25 °C, 1–3 h)	[17,221]
Selenoamides	NaHSe (1.0 *Eq.*), EtOH, 1 h *	80–95% (EtOH, Cr(CO)_6_, H_2_N–R, Se, NaBH_4_, 25 °C, 1–3 h)	[200,203]
Selenium nanoparticles	80–98% (plant/microbial biosynthesis)	50–70% (chemical reduction with NaBH_4_)	[222,223]

Notes: * Selenoamide is an intermediate; the yield of the conventional route for selenoamide was not reported by Makhaeva et al. [203].

## Data Availability

No new data were created or analyzed in this study. Data sharing is not applicable to this article.

## Supplementary Materials

The following supporting information can be downloaded at https://www.mdpi.com/article/10.3390/molecules31152674/s1: Table S1: Overview of organoselenium compounds: chemical structures, biological activities, study types, molecular target and pharmacological test.

## List of Abbreviations

API	Active pharmaceutical ingredient
C_6_H_8_O_7_	Citric acid
GAPI	Green Analytical Procedure Index
GPx	Glutathione peroxidases
GSH	Glutathione
ILs	Ionic liquids
LCA	Life Cycle Assessment
LDL	Low-density lipoprotein
MAD	Multi-wavelength anomalous dispersion
MMPP	Magnesium monoperoxyphthalate
NCL	Native chemical ligation
NCS	*N*-chlorosuccinimide
NEXAFS	Near-edge X-ray absorption fine structure
NMP	*N*-methyl-2-pyrrolidone
ROS/RNS	Reactive oxygen and nitrogen species
SAD	Single-wavelength anomalous dispersion
scCO_2_	Supercritical carbon dioxide
SeCys	Selenocysteine
SeMet	Selenomethionine
SeNPs	Selenium nanoparticles
SPPS	Solid-phase peptide synthesis
TrxR	Thioredoxin reductase
