# Supplementary material for: Progress in the Synthesis of Organoselenium Compounds: Conventional Routes Versus Green Approaches
Source: Molecules. 2026 Jul 31;31(15):2674. doi: 10.3390/molecules31152674 (PMC13467645; doi:10.3390/molecules31152674)
Supplement: Supplementary file 1 [file molecules-31-02674-s001.zip › molecules-4442168-supplementary.pdf]

Progress in the Synthesis of Organoselenium Compounds: Conventional Routes Versus Green Approaches

Chintankumar Padariya * and Anita Kornicka

Department of Chemical Technology of Drugs, Faculty of Pharmacy, Medical University of Gdańsk, Al. Gen. J. Hallera 107, 80-416 Gdańsk, Poland;

anita.kornicka@gumed.edu.pl

* Correspondence: chintankumar.padariya@gumed.edu.pl; Tel.: +48-58-349-19-53

Table S1*: Overview of organoselenium compounds: chemical structures, biological activities, study types, molecular target and pharmacological test.*

Compounds	Biological Activity	Study Type	Molecular Target	References*
		**Selenium amino acid analogues**		
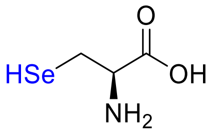<br>Selenocysteine	Anticancer	*In vitro*<br>(MTT cell viability assay using cancerous and non-cancerous cell lines; ROS-generation studies; flow cytometry with annexin V staining; single-cell gel electrophoresis for DNA-strand breaks)	ROS-mediated apoptosis and DNA damage-mediated apoptosis	[28]
	Anticancer	*In vitro*<br>(MTT cell viability assay using human glioma cells; flow cytometric assay; cell cycle analysis; DCFH-DA scavenging assay; western blot analysis)	ROS-mediated DNA damage; MAPK and Akt signaling pathways	[29]
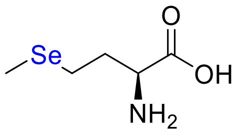<br>Selenomethionine	Anti-atherosclerotic; vasculoprotective	*In vivo*<br>(ApoE mouse model of atherosclerosis)<br>*Ex vivo*<br>(aortic-vessel function analysis)	GPx-antioxidant defence system; M1 inflammatory type macrophages; vessel function	[53]
	Anti-ferroptotic; neuroprotective	*In vitro*<br>(MTT cell viability assay using N27 cells derived from E12 rat mesencephalic tissue; erastin- and RSL3-induced ferroptosis; protein assays)<br>*In vivo*<br>(MCAO-induced focal cerebral ischemia–reperfusion mouse model)	GPx/GPX4-mediated ferroptosis	[54]

Table S1: Continued.

Compounds	Biological Activity	Study Type	Molecular Target	References*
		**Seleninic acid analogues**		
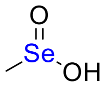 Methylseleninic acid	Anticancer	*In vitro* (TM6 mouse mammary epithelial cells; kinase assays and protein-kinase screening)	Cyclin E–CDK2 and cyclin E-associated kinases	[35]
	Anticancer	*In vitro* (cell-based viability test in 2D and 3D Panc-1 cultures; western blotting analysis; cell cycle analysis; clonogenic assay)	Entosis; CDC42 and CD29 signaling pathways	[36]
		**Selenols**		
$H_3C-Se-H$ Monomethyl selenol	Anticancer	*In vitro* (cell-based viability assay using H157 lung carcinoma cell assay; Trx/TrxR and GPx activity measurements; ROS production measurement; clonogenic assay)	Trx/TrxR and Grx/GSH systems	[37]
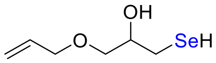 1-(allyloxy)-3-hydroselenopropan-2-ol	Carbonic-anhydrase inhibition	*In vitro* (enzymatic inhibition assays for hCA I, hCA II, and hCA VII)	hCA I, hCA II, and hCA VII	[38]

Table S1: Continued.

Compounds	Biological Activity	Study Type	Molecular Target	References*
		**Heterocyclic selenides**		
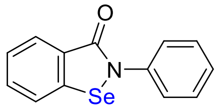<br>Ebselen	Anti-inflammatory	*In vitro*<br>(LPS-stimulated rat Kupffer cell assay; NO/TNF-α measurements and NF-κB/JNK signaling analyses)	JNK and NF-κB signaling pathways; COX-2	[55]
	Antibacterial	*In vitro*<br>(spectrophotometric analysis of MDR *S. aureus* LT-1; growth inhibition; DTNB–TrxR, and ROS assays)<br>*In vivo*<br>(rat model of *S. aureus* LT-1 skin-wound infection)	Bacterial TrxR/redox homeostasis; pro-inflammatory cytokines (TNF-α, IL-6, and IL-1β)	[39]
	Antiviral	*In vitro*<br>(enzyme assay; inhibition assay; molecular modeling; Ub-AMC fluorogenic assay)	SARS-CoV-2 papain-like protease (PL^{pro} CoV2); irreversible covalent inhibition involving catalytic Cys111	[56]
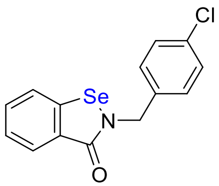<br>2-(4-Chlorobenzyl)benzo[*d*][1,2]selenazol-3(*2H*)-one	Antiviral	*In vitro*<br>(M^{pro} enzymatic inhibition and cell-based antiviral assays using SARS-CoV-2-infected Vero E6 cells)	SARS-CoV-2 main protease (M^{pro}/3CLpro)	[57]

***Table S1**: Continued.*

Compounds	Biological Activity	Study Type	Molecular Target	References*
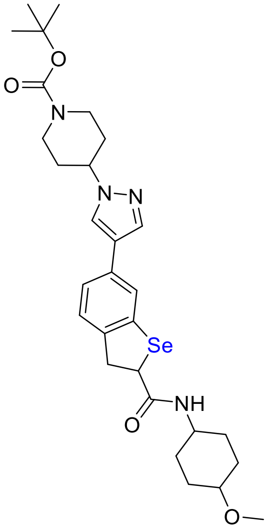 4-(4-(2-((4-methoxycyclohexyl)carbamoyl)benzo[*b*]selenophen-5-yl)-1*H*-pyrazol-1-yl)piperidine-1-carboxylate	Anti-inflammatory, antioxidant, antifibrotic	*In vitro* (cell-viability and cytotoxicity assays using MRC-5 cells; TGF-β1-induced cellular fibrotic model in MRC-5 cells; pro-inflammatory cytokine assays; measurement of intracellular ROS, SOD, GSH, and MDA; Western blot and mRNA analysis of fibrosis markers in TGF-β-induced MRC-5 cells; immunofluorescence staining; enzymatic assay; CETSA assay) *In vivo* (BLM-induced pulmonary fibrosis mouse model (C57BL/6); histopathological analysis, serum/BALF cytokine measurements; Western blot analysis; pharmacokinetic analyses)	Keap1/Nrf2/HO-1/NQO1, NF-κB p65, TGF-β/Smads, YAP/TAZ-TEAD signaling pathways	[40]
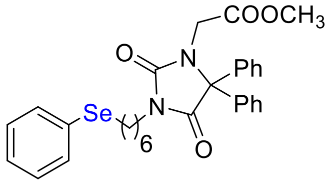 Methyl 2-(2,5-dioxo-4,4-diphenyl-3-(6-(phenylselanyl)hexyl) imidazolidin-1-yl)acetate	Anticancer	*In vitro* (cell-based cytotoxic and antiproliferative assays; ABCB1 efflux-modulation assay)	ABCB1 transporter (P-glycoprotein efflux pump)	[41]

***Table S1**: Continued.*

Compounds	Biological Activity	Study Type	Molecular Target	References[*]
		**Selenides and diselenides**		
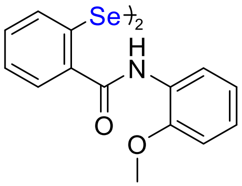 Bis[2-(2-methoxyphenylcarbamoyl)phenyl] diselenide	Antiviral	*In vitro* (enzyme assay; inhibition assay; molecular modeling; Ub-AMC fluorogenic assay)	SARS-CoV-2 papain-like protease (PL^{pro} CoV2); irreversible covalent inhibition involving catalytic Cys111	[56]
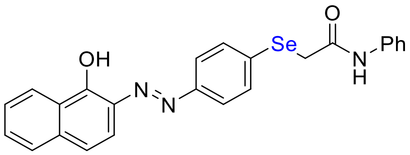 2-((4-((1-hydroxynaphthalen-2-yl)diazenyl)phenyl)selanyl)-*N*-phenylacetamide	Anticancer; antimicrobial; antioxidant	*In vitro* (MTT cell viability assay using MCF-7 and HepG2 cells; agar diffusion assays using *E. coli*, *S. aureus*, and *C. albicans*; DPPH and ABTS radical scavenging assays)	Not indicated	[58]
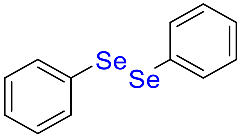 Diphenyl diselenide	Anti-atherogenic	*In vitro* (DCFH-DA radical scavenging assay; enzymatic, immunofluorescence, cell-viability assays)	NF-κB/MMP-9/redox signaling pathways	[42]

Table S1: Continued.

Compounds	Biological Activity	Study Type	Molecular Target	References*
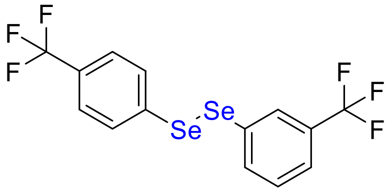<br>3-(trifluoromethyl)-diphenyldiselenide<br>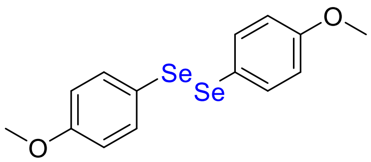<br>4-methoxydiphenyldiselenide	Anticancer	*In vitro*<br>(MTT assay for viability of HT-29 cancer cells; flow cytometry and qPCR analyses)	Caspase-dependent and caspase-independent apoptosis	[43]
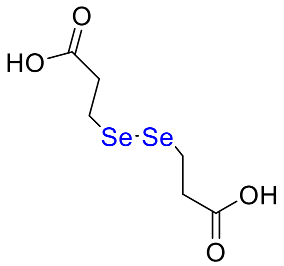<br>3,3′-diselenodipropionic acid	Radioprotective	*In vivo*<br>(γ-irradiated Swiss albino mouse model)	Antioxidant enzymes; apoptosis; attenuation of DNA damage	[44]

***Table S1**: Continued.*

Compounds	Biological Activity	Study Type	Molecular Target	References*
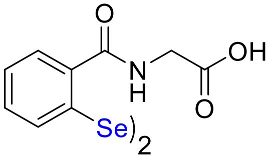 2,2-((2,2′-diselanediylbis(benzoyl))bis(azanediyl))diacetic acid	Antiviral	*In vitro* and *in silico* (antiviral assays and molecular modeling)	HIV-1 NCp7 zinc-finger domain; Gag processing	[45]
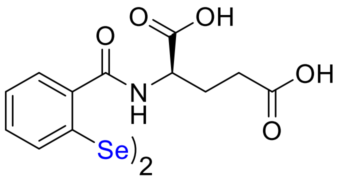 (2*S*,2′*S*)-2,2′-((2,2′-diselanediylbis(benzoyl))bis(azanediyl))- dipentanedioic acid				
**Selenooxides**				
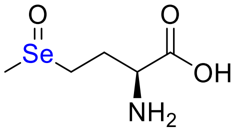 Selenomethionine selenoxides	Catalytic scavenging of MPO-derived oxidants	*In vitro* (biochemical oxidant-scavenging assays using TMB; scavenging of MPO- and neutrophil-derived oxidants)	TrxR/NADPH and GSR/NADPH systems	[46]
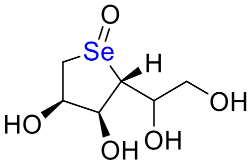 1,4-Anhydro-4-seleno-D-talitolselenoxide				

***Table S1**: Continued.*

Compounds	Biological Activity	Study Type	Molecular Target	References*
		**Selenones**		
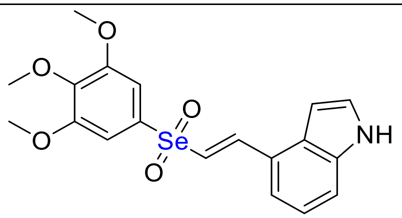 Vinyl selenone	Anticancer	*In vitro* (MTT cell viability assay in cancer cell lines; cell cycle and apoptosis analysis; immunofluorescence-based microtubule assays) *In vivo* (liver cancer allograft mouse model)	Tubulin polymerization; G2/M-phase cell-cycle; apoptosis	[47]
		**Selenoureas**		
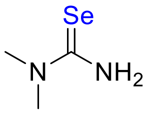 *N*,*N*-dimethylselenourea	Antioxidant	*In vitro* (cell-based cytotoxic assay - microtiter tetrazolium method with the use of HaCaT, HEL, and HEp-2 cells; polymorphonuclear leukocytes real-time kinetic chemiluminescence method; laser screening microscopy - visualization of p47 phagocyte oxidase (p47[phox]))	O_2^{-}- scavenging	[48]
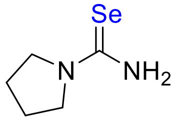 1-selenocarbamoylpyrrolidine				

Table S1*: Continued.*

Compounds	Biological Activity	Study Type	Molecular Target	References[*]
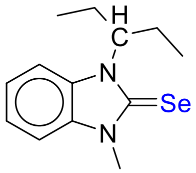 1-isopropyl-3-methylbenzimidazole-2-selenone	Antifungal; antioxidant	*In vitro* (agar reducing procedure for antifungal activity; DPPH radical scavenging assay)	Not indicated	[49]
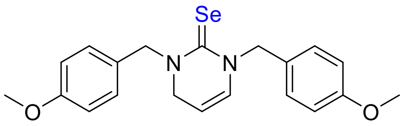 1, 3-di-*p*-methoxybenzylpyrimidine-2-selenone				
**Seleno-urea-containing dihydropyrrol-2-one derivatives**				
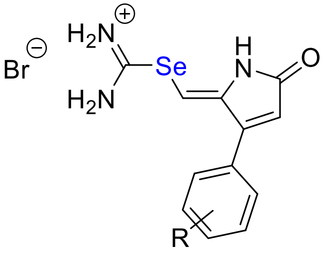 R = H, F, Cl, Br, OCH_3 Selenourea-containing dihydropyrrol-2-one (DHP) derivatives	Antibacterial	*In vitro* (quorum-sensing inhibition using the *P. aeruginosa* MH602 P_{las}B:gfp reporter strain; broth microdilution assay with the use of *S. aureus* SA38 and *E. coli* K-12)	LasR quorum-sensing system	[50]

Table S1*: Continued.*

Compounds	Biological Activity	Study Type	Molecular Target	References*
		**Selenoamides**		
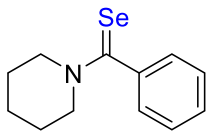<br>*N*-(phenylselenocarbonyl)-piperidine<br>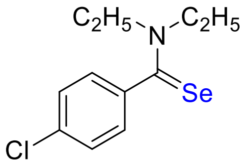<br>*N*,*N*-diethyl-4-chloroselenobenzamide	Antioxidant	*In vitro*<br>(cell-based cytotoxic assay - microtiter tetrazolium method with the use of HaCaT, HEL, and HEp-2 cells; polymorphonuclear leukocytes real-time kinetic chemiluminescence method; laser screening microscopy - visualization of p47 phagocyte oxidase ($p47^{phox}$))	O_2^-- scavenging	[48]
		**Selenocynates**		
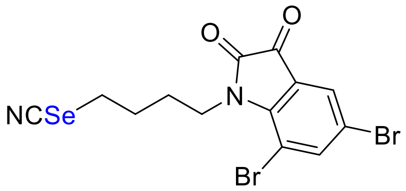<br>5,7-dibromo-*N*-(4’-selenocyanatobutyl)isatin	Anticancer	*In vitro*<br>(cell-viability and proliferation assays using A549, HT-29, MCF-7, and UACC903 cells; cell cycle analysis; caspase-3/7, TUNEL, and tubulin-polymerization assays; western blotting)	Tubulin polymerization and the Akt signaling pathway	[51]

***Table S1**: Continued.*

Compounds	Biological Activity	Study Type	Molecular Target	References*
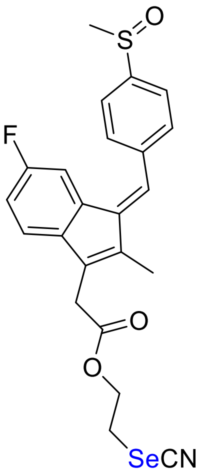<br>2-selenocyanatoethyl (*Z*)-2-(5-fluoro-2-methyl-1-(*4*-(methylsulfinyl)benzylidene)-1*H*-inden-3-yl)acetate	Anti-neuroinflammatory	*In vitro*<br>(cell-viability assay using BV2 microglial cells; DCFH-DA radical scavenging assay; RT-qPCR; western blot analysis; assay IL-18 and IL-1β by ELISA)	NOX2/NLRP3 signaling pathway	[64]
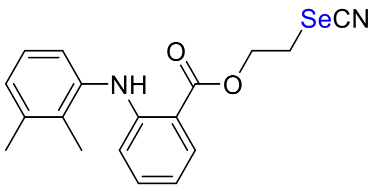<br>2-selenocyanatoethyl 2-((2,3-dimethylphenyl)amino)benzoate				
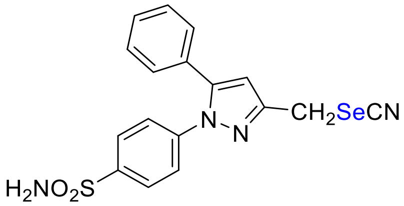<br>Selenocoxib-1	Anticancer	*In vitro*<br>(MTT cell viability assay in PAIII and PC-3M prostate cancer cells; Western blotting; enzyme assays)<br>*In vivo*<br>(short-term PAIII transplantable tumor model in Lobund–Wistar rats)	COX-2 expression; HIF-1α-mediated hypoxic signaling; p-Akt-mediated cell-survival signaling; anti-apoptotic protein Bcl-2	[52]

Note: * - The main text of the manuscript contains full list of references.
